# Macrophage CREBZF Orchestrates Inflammatory Response to Potentiate Insulin Resistance and Type 2 Diabetes

**DOI:** 10.1002/advs.202306685

**Published:** 2024-01-29

**Authors:** Yuxiao Liu, Weitong Su, Zhengshuai Liu, Zhimin Hu, Jiaxin Shen, Zengpeng Zheng, Dong Ding, Wei Huang, Wenjing Li, Genxiang Cai, Shuang Wei, Ni Li, Xia Fang, Hong Li, Jun Qin, Haibing Zhang, Yichuan Xiao, Yan Bi, Aoyuan Cui, Chunxiang Zhang, Yu Li

**Affiliations:** ^1^ CAS Key Laboratory of Nutrition Metabolism and Food Safety Shanghai Institute of Nutrition and Health University of Chinese Academy of Sciences Chinese Academy of Sciences Shanghai 200031 China; ^2^ Department of Endocrinology and Metabolism The Affiliated Hospital of Southwest Medical University Metabolic Vascular Diseases Key Laboratory of Sichuan Province Luzhou Sichuan 646000 China; ^3^ CAS Key Laboratory of Tissue Microenvironment and Tumor Shanghai Institute of Nutrition and Health University of Chinese Academy of Sciences Chinese Academy of Sciences Shanghai 200031 China; ^4^ CAS Key Laboratory of Computational Biology Shanghai Institute of Nutrition and Health Chinese Academy of Sciences Shanghai 200031 China; ^5^ Affiliated Drum Tower Hospital Medical School of Nanjing University Nanjing Jiangsu 210008 China; ^6^ Metabolic Vascular Disease Key Laboratory of Sichuan Province The Affiliated Hospital of Southwest Medical University Key Laboratory of Medical Electrophysiology Ministry of Education Southwest Medical University Luzhou 646000 China

**Keywords:** adipose tissue macrophage, chronic inflammation, CREBZF, NF‐κB, type 2 diabetes

## Abstract

Chronic adipose tissue inflammation accompanied by macrophage accumulation and activation is implicated in the pathogenesis of insulin resistance and type 2 diabetes in humans. The transcriptional coregulator CREBZF is a key factor in hepatic metabolism, yet its role in modulating adipose tissue inflammation and type 2 diabetes remains elusive. The present study demonstrates that overnutrition‐induced CREBZF links adipose tissue macrophage (ATM) proinflammatory activation to insulin resistance. CREBZF deficiency in macrophages, not in neutrophils, attenuates macrophage infiltration in adipose, proinflammatory activation, and hyperglycemia in diet‐induced insulin‐resistant mice. The coculture assays show that macrophage CREBZF deficiency improves insulin sensitivity in primary adipocytes and adipose tissue. Mechanistically, CREBZF competitively inhibits the binding of IκBα to p65, resulting in enhanced NF‐κB activity. In addition, bromocriptine is identified as a small molecule inhibitor of CREBZF in macrophages, which suppresses the proinflammatory phenotype and improves metabolic dysfunction. Furthermore, CREBZF is highly expressed in ATM of obese humans and mice, which is positively correlated with proinflammatory genes and insulin resistance in humans. This study identifies a previously unknown role of CREBZF coupling ATM activation to systemic insulin resistance and type 2 diabetes.

## Graphical abstract

This study identifies mechanisms of macrophage and adipocyte crosstalk during obesity. In response to overnutrition, CREBZF in macrophages is activated and aggravates inflammation leading to impaired insulin signaling. CREBZF competitively inhibits the binding of newly synthesized IκBα to p65, and enhances p65 nuclear localization and transcriptional activity. Pharmacological and genetic inhibition of CREBZF attenuates hyperglycemia and type 2 diabetes.

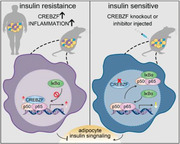

## 1 Introduction

With the increased prevalence rate of type 2 diabetes (T2DM) worldwide, it is urgent to develop new and improved therapeutic strategies.^[^ ^1^ ^]^ Insulin resistance that is associated with obesity plays a key role in the development of T2DM. Obesity causes changes in the content and activation state of various immune cell types, triggering and promoting low‐grade tissue inflammation and insulin resistance.^[^ ^2^ ^]^ Among the immune cells, macrophages are critical and decisive in orchestrating inflammation responses.^[^ ^3^ ^]^ Macrophages infiltrate adipose tissue during obesity and respond to environmental factors, exhibiting a spectrum of functionally distinct activation phenotypes.^[^ ^4^ ^]^ Recent studies suggest that inflammatory cytokines directly or indirectly inhibit insulin signaling.^[^ ^5^ ^]^ Thus, identifying the triggers that regulate proinflammatory pathways and cytokine production is vital for understanding inflammation and insulin resistance.

CREB/ATF bZIP transcription factor (CREBZF) has recently emerged as an important transcriptional coregulator involved in hepatic metabolism and cell growth.^[^ ^6^, ^7^ ^]^ Recent studies demonstrate that hyperactivation of CREBZF accelerates the pathogenesis of NAFLD and NASH. In hepatocytes, CREBZF is induced by insulin and inhibits the transcription of insulin‐induced genes (Insigs), leading to increased hepatic steatosis.^[^ ^8^ ^]^ CREBZF increases hepatic stellate cell activation and fibrosis by stimulating osteopontin in NASH.^[^ ^9^ ^]^ Moreover, CREBZF inhibits liver regeneration by repressing STAT3.^[^ ^10^ ^]^ Although transcriptional networks of CREBZF in liver metabolism and bZIP proteins are involved in inflammation, such as the C/EBP family, whether CREBZF regulates inflammatory pathways and insulin resistance remains unknown.

Stimulus‐regulated transcription factors were found to promote the expression of specific genes that dictate the functional polarization of macrophages, such as NF‐κB, AP‐1, STAT, and IRF families.^[^ ^11^ ^]^ Notably, coregulators may regulate transcription by preventing or increasing the activity of transcriptional factors, which become important in light of disease‐associated alterations. However, the role of coregulators under specific pathological conditions remains largely unknown. Therefore, it is urgently needed to explore and characterize novel coregulators that are involved in the inflammatory pathway and modulate macrophage activation, particularly in the context of obesity‐related chronic inflammation and insulin resistance.

This study provides insights into mechanisms that accelerate the progression of obesity‐associated inflammation toward insulin resistance and T2DM and further demonstrates the potential of targeting transcriptional coregulator of ATM for treating insulin resistance. Gain‐ and loss‐of‐function approaches demonstrate that CREBZF‐mediated regulation of the NF‐κB signaling couples inflammatory signals to metabolic homeostasis in obesity. These in vivo and in vitro studies demonstrate that 1) CREBZF deficiency in macrophages improves insulin sensitivity via the crosstalk between ATM and adipocytes; 2) CREBZF promotes NF‐κB signaling by competitively inhibiting the binding of IκBα to p65; 3) Pharmacological inhibition of CREBZF corrects ATM activation and type 2 diabetes in mice; 4) CREBZF is highly expressed in ATM of obese humans and mice, which is correlated with proinflammation genes and insulin resistance in humans.

## 2 Results

### 2.1 Hyperglycemia and Insulin Resistance Are Improved in Myeloid CREBZF Deficient Mice Fed with HFHS Diet

**Figure 1:**
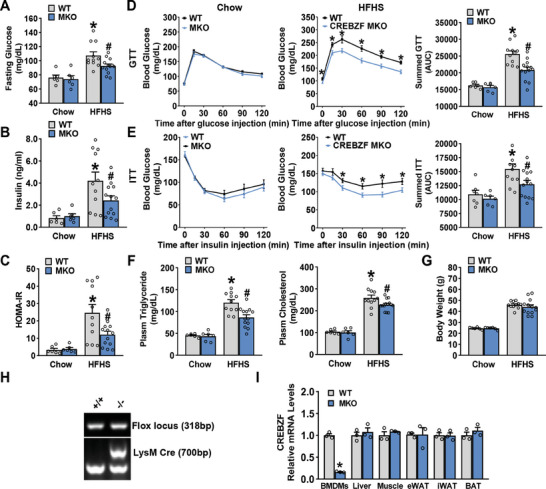
Myeloid CREBZF deficiency protected mice from diet‐induced hyperglycemia and insulin resistance. Male CREBZF MKO and WT mice at 8 weeks old were fed with HFHS diet for 16 weeks. A) Fasting blood glucose and B) plasma insulin levels were assessed. C) HOMA‐IR. D) The glucose tolerance test (1 g kg^−1^) and respective area under the curve were performed. E) Insulin tolerance test (1 U kg^−1^) and respective area under the curve were performed. F) Plasma triglyceride and cholesterol levels were measured. G) Body weight. A–G) Chow diet group, *n* = 6–7. HFHS diet group, *n* = 11–13. ^*^ *p* < 0.05 versus WT mice fed with chow diet; ^#^ *p* < 0.05 versus WT mice fed with HFHS diet. H) PCR amplification of LysM‐Cre alleles for genotyping. I) The knockout efficiency of CREBZF was verified. qPCR analysis of CREBZF mRNA expression levels in BMDMs, liver, muscle, and various adipose tissues, *n* = 3. ^*^ *p* < 0.05 versus WT mice fed with chow diet.

To address the role of macrophage CREBZF in regulating metabolic dysfunction, myeloid‐specific CREBZF knockout (CREBZF MKO) mice were generated. Given the similar ratio of macrophages, neutrophils, and dendritic cells in the bone marrow and blood, CREBZF deficiency in myeloid cells did not affect the development and maturation of myeloid cells (Figure S1A,B, Supporting Information). Additionally, myeloid‐specific CREBZF deficiency mice exhibited normal T cell development in the thymus as well as the maturation and activation of peripheral lymphoid cells (Figure S1C–E, Supporting Information). Collectively, CREBZF MKO mice showed no defective developmental phenotypes and had insignificant differences in metabolic characteristics when fed a chow diet (**Figure** **1**A–G). Strikingly, CREBZF MKO mice were resistant to high‐fat, high‐sucrose (HFHS) diet‐induced metabolic deterioration (Figure 1A–F), which was widely used as a diet‐induced diabetogenic model.^[^ ^8^ ^]^ Compared with WT littermates, fasting glucose, serum insulin, and HOMA‐IR were decreased in CREBZF MKO mice fed with HFHS diet (Figure 1A–C), indicating improved insulin sensitivity. CREBZF MKO mice also exhibited markedly improved blood glucose control and insulin tolerance (Figure 1D,E). Significant decreases in serum triacylglycerol and cholesterol were observed in HFHS diet‐fed CREBZF MKO mice, while body weight was unchanged (Figure 1F,G). The knockout efficiency was verified by real‐time PCR and immunoblotting analysis (Figure 1H,I; Figure S1F, Supporting Information). These data show that myeloid CREBZF deficiency ameliorates obesity‐associated insulin resistance, suggesting CREBZF in myeloid cells couples overnutrition to obesity‐associated metabolic dysfunctions.

### 2.2 Macrophage CREBZF Is Responsible for the Reduced ATM Activation, Inflammation, and Insulin Resistance in CREBZF MKO Mice

**Figure 2:**
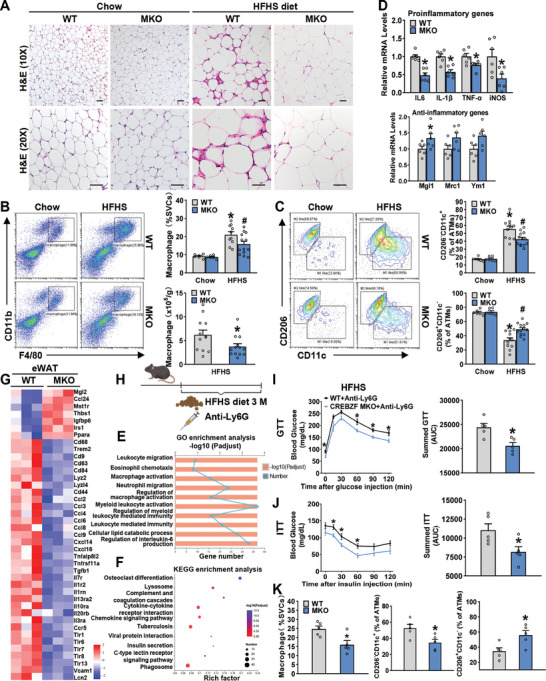
Myeloid CREBZF deficiency alleviated adipose tissue inflammation in HFHS‐fed mice. Male CREBZF MKO and WT mice at 8 weeks old were fed with an HFHS diet for 16 weeks. A) H&E staining of eWAT. Scale bars, 50 µm. B) Representative flow cytometry analysis and quantification of the F4/80^+^CD11b^+^ macrophages in SVF from eWAT. C) Representative flow cytometry analysis and quantification of the CD11c^+^CD206^−^ and CD11c^−^CD206^+^ subset of total macrophages from eWAT. B,C) Chow diet group, *n* = 6–7. HFHS diet group, *n* = 11–13. ^*^ *p* < 0.05 versus WT mice fed with chow diet; ^#^ *p* < 0.05 versus WT mice fed with HFHS diet. D) qPCR analysis indicating mRNA abundance of proinflammatory and anti‐inflammatory genes in eWAT SVF isolated from CREBZF MKO and WT mice fed with HFHS diet. *n* = 6. ^*^ *p* < 0.05. eWAT from CREBZF MKO and WT mice fed with HFHS diet were performed RNA‐seq. *n* = 3. GO enrichment analysis (E) and KEGG enrichment analysis (F) showed the most significantly enriched biological process. G) Heatmap showed the immune system process related to differently expression genes. H) The model of neutrophil depletion assay. CREBZF MKO and WT mice were deleted of neutrophils with anti‐Ly6G antibody (1A8) and fed with HFHS diet for 12 weeks. I) Glucose tolerance test (1 g kg^−1^) and (J) insulin tolerance test were performed in mice lacking neutrophils. K) Flow cytometry analysis of macrophages in eWAT from mice lacking neutrophils. I–K) *n* = 5, ^*^ *p* < 0.05 versus WT+Anti‐Ly6G.

Next, we addressed the role of macrophage CREBZF in obesity‐associated inflammation induced by HFHS diet. The crown‐like structures (CLS) are histologic hallmarks of the adipose tissue inflammatory process. Compared with WT littermates, reduced CLS was observed in epididymal adipose tissue (eWAT) of CREBZF MKO mice fed with a HFHS diet (**Figure** **2**A). Consistently, the total number of F4/80^+^CD11b^+^ macrophages in eWAT of CREBZF MKO mice was significantly lower (Figure 2B). Classically activated macrophages (M1‐like) represent a proinflammatory type, which secret a range of proinflammatory mediators and exert adverse effects on insulin sensitivity.^[^ ^12^ ^]^ Whereas alternatively activated macrophages (M2‐like) primarily exhibit an anti‐inflammatory state involved in the resolution of inflammation and adaptive thermogenesis.^[^ ^13^ ^]^ A significant decrease in the percentage of CD11c^+^CD206^−^ proinflammatory (M1‐like) macrophages was detected in eWAT of CREBZF MKO mice, concomitant with an increase in the percentage of CD11c^−^CD206^+^ anti‐inflammatory (M2‐like) macrophages (Figure 2C). Increased M1‐/M2‐like macrophage ratio has been described to be responsible for metabolic disorders in obese mice.^[^ ^14^ ^]^ Strikingly, CREBZF deficiency improved the imbalance of the M1‐/M2‐like macrophage ratio of eWAT (Figure S2D, Supporting Information). In line with these findings, SVF isolated from eWAT of CREBZF MKO mice expressed lower levels of M1‐like macrophage‐related genes obviously, along with higher levels of M2‐like macrophage‐related genes (Figure 2D). Reduced infiltration of macrophages was also observed in inguinal adipose tissue (iWAT) of CREBZF MKO mice (Figure S2A, Supporting Information). Although the percentages of macrophage subpopulations and expression of inflammatory genes showed no significant changes in SVF isolated from iWAT of CREBZF MKO mice, a trend toward attenuated inflammation was observed (Figure S2B,C, Supporting Information). Moreover, to generally understand the effects of myeloid CREBZF deficiency on adipose tissue homeostasis, a genome‐wide transcriptomic analysis by RNA sequencing of eWAT in HFHS‐fed mice was performed. A total of 1410 differentially expressed genes (DEGs) were identified between WT and CREBZF MKO mice (Figure S3A, Supporting Information). Among the top differentially regulated pathways derived from Gene Ontology (GO) and Kyoto Encyclopedia of Genes and Genomes analysis (KEGG) of DEGs, most pathways were involved in immune response (Figure 2E,F). A heatmap analysis showed multiple inflammation‐related DEGs, indicating myeloid CREBZF deficiency altered the transcriptome landscape in adipose tissue inflammation (Figure 2G). The activation of proinflammatory cells in adipose tissue leads to adipocyte death and eventual fibrosis and is a hallmark of adipose tissue dysfunction and obesity‐associated insulin resistance.^[^ ^15^ ^]^ As shown in Figure S3B (Supporting Information), decreased fibrosis‐related genes were observed in eWAT from CREBZF MKO mice, indicating improved extracellular matrix (ECM) remodeling. Expression levels of representative marker genes for other immune cells present in the SVF of eWAT in WT and CREBZF MKO mice fed with HFHS diet were analyzed. As shown in the revised Figure S3C (Supporting Information), expression levels of Foxp3, a marker for Treg cells, were significantly upregulated in SVF from CREBZF MKO mice. Moreover, the diet‐induced hepatic steatosis and insulin resistance in the liver were also improved in CREBZF MKO mice (Figure S3D,E, Supporting Information). These data suggest that myeloid CREBZF may play essential roles in the regulation of adipose tissue inflammation and dysfunction.

Additionally, it has been reported that LysM‐Cre is expressed in both macrophages and neutrophils.^[^ ^16^ ^]^ To assess whether the effects observed in CREBZF MKO mice are dependent on neutrophils, a neutrophil depletion assay in vivo by injecting anti‐mouse Ly6G antibodies was performed (Figure 2H; Figure S4A–D, Supporting Information). In the absence of neutrophils, CREBZF deficiency in myeloid cells remains the effects of attenuated HFHS diet‐induced glucose intolerance, insulin resistance, and inflammation (Figure 2I–K). Collectively, these results establish that CREBZF in macrophages is responsible for the regulation of inflammation and metabolic homeostasis.

Subsequently, the causal relationship between attenuation of inflammation and improvement of metabolism by myeloid CREBZF deficiency was investigated. CREBZF MKO mice and their WT littermates were fed with HFHS diet for eight weeks. There was no difference in glucose tolerance and insulin sensitivity (Figure S5A–C, Supporting Information). However, CREBZF MKO mice exhibited a significantly diminished inflammatory environment in eWAT, such as reduced M1‐like proportions and decreased proinflammatory gene levels (Figure S5D,E, Supporting Information). The finding indicates that the attenuation of inflammation by CREBZF deficiency preceded the improvement of glucose tolerance and insulin sensitivity.

### 2.3 Improved Inflammation and Insulin Sensitivity by CREBZF Deficiency Are Mediated by the Crosstalk Between ATM and Adipocytes

**Figure 3:**
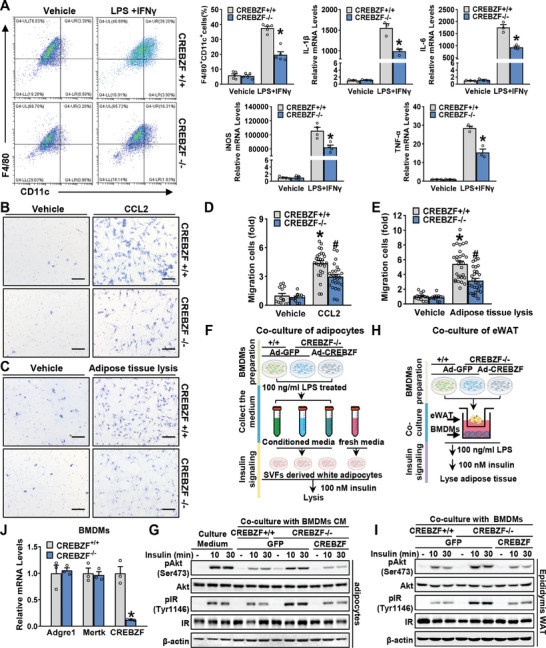
CREBZF deficiency of macrophages enhanced adipocyte insulin sensitivity through repression of inflammatory signaling. A) BMDMs from CREBZF MKO and WT mice were stimulated with 100 ng mL^−1^ LPS and 20 ng mL^−1^ IFNγ for 24  h. The percentages of F4/80^+^CD11c^+^ M1‐like macrophages were assessed by flow cytometry and M1‐like marker genes were determined by qPCR. *n* = 3–5. ^*^ *p* < 0.05 versus CREBZF^+/+^ BMDMs treated with vehicle; ^#^ *p* < 0.05 versus CREBZF^+/+^ BMDMs treated with LPS and IFNγ. (B–E) Chemotaxis of BMDMs from WT and CREBZF MKO mice, treated with the chemoattractant CCL2 or fresh adipose tissue lysate from obese mice. B,C) Representative filters. Scale bars, 50 µm. D,E) Quantitation of data with the total number of cells migrated, normalized by CREBZF^+/+^ BMDMs treated with vehicle. Vehicle group, *n* = 12–14, chemoattractant treated group, *n* = 27–29. ^*^ *p* < 0.05 versus CREBZF^+/+^ BMDMs treated with vehicle; ^#^ *p* < 0.05 versus CREBZF^+/+^ BMDMs treated with chemoattractant as indicated. F) Schematic depicting the white adipocytes derived from SVF treated with LPS‐stimulated conditioned medium. G) Immunoblot analysis of white adipocytes treated with LPS‐stimulated conditioned medium from CREBZF^+/+^ and CREBZF^−/−^ BMDMs, followed by a treatment of 100 nm insulin for 10 min or 30 min. H) Schematic depicting the coculture system of BMDMs and white adipose tissues. I) Immunoblot analysis of white adipose tissues cocultured with BMDMs treated as indicated. J) Relative mRNA abundance of Adgre1, Mertk, and CREBZF in BMDMs differentiated for 7 days. *n* = 3. ^*^ *p* < 0.05 versus WT mice fed with chow diet.

Next, we explored how CREBZF regulates macrophages to influence metabolic homeostasis and insulin resistance. Bone‐marrow‐derived macrophages (BMDMs) are incubated with LPS and IFNγ to mimic M1 polarization,^[^ ^17^ ^]^ which induces the expression of prototypical target genes that characterize the M1 phenotype. Compared to CREBZF^+/+^ BMDMs treated with LPS and IFNγ, CREBZF deficient BMDMs demonstrated a decrease in F4/80^+^CD11c^+^ cell number and proinflammatory genes expression, including IL‐1β, IL‐6, TNFα, and iNOS (**Figure** **3**A). Notably, the loss of CREBZF in myeloid cells reduced the number of macrophages in that tissue (Figure 2A,B). To further investigate whether the chemotactic responses of macrophages were affected by CREBZF deficiency, chemotactic migration assays were performed using a transwell chamber. As shown in Figure 3B–E, CREBZF^−/−^ BMDMs exhibited reduced chemotactic activity responding to both chemoattractant CCL2 (MCP‐1) and fresh adipose tissue lysate of obese mice, which contained a mixture of various chemotactic factors. These results demonstrate that CREBZF is a positive regulator of the proinflammatory functions of macrophages.

Given the reduced proinflammatory functions of macrophages caused by CREBZF deficiency, we further determine the effects of macrophage CREBZF on insulin sensitivity in adipocytes. The differentiated white adipocytes derived from SVF were incubated with a conditioned medium collected from macrophages. The sensitivity of insulin signaling in adipocytes was measured (Figure 3F). Insulin‐induced phosphorylation of Akt and IR were attenuated when treated with a conditioned medium from LPS‐stimulated CREBZF^+/+^ BMDMs. Interestingly, the inhibitory effects were largely attenuated when treated with a conditioned medium from CREBZF^−/−^ BMDMs. Furthermore, conditioned medium from Ad‐CREBZF‐treated CREBZF^−/−^ BMDMs reversed these effects, suggesting that insulin sensitivities of adipocytes are negatively regulated by CREBZF in macrophages (Figure 3G). Next, the BMDMs of WT and MKO mice were cocultured with pieces of intact white adipose tissue from WT lean mice. As shown in Figure 3H, the activity of insulin signaling in white adipose tissues was reduced when co‐culturing with CREBZF^+/+^ BMDMs, which was reversed by CREBZF deficiency (Figure 3I). Furthermore, when the BMDMs were incubated with interleukin‐4 (IL‐4) to direct them to an M2‐like phenotype, mild inductions of CD11b^+^CD206^+^ cell numbers of M2‐like macrophages and expression levels of anti‐inflammatory genes were observed in CREBZF deficient BMDMs (Figure S6, Supporting Information). These observations are consistent with the effects of CREBZF on proinflammatory phenotypes of macrophage. Notably, CREBZF deficiency does not affect the development of BMDMs as indicated by the comparable levels of Adgre1 and Mertk at day 7 of differentiation (Figure 3J). Thus, these results support the notion that CREBZF deficiency results in reduced proinflammatory macrophage activation and enhanced insulin sensitivity in adipocytes.

### 2.4 CREBZF Regulates NF‐κB Pathway in the Macrophage

**Figure 4:**
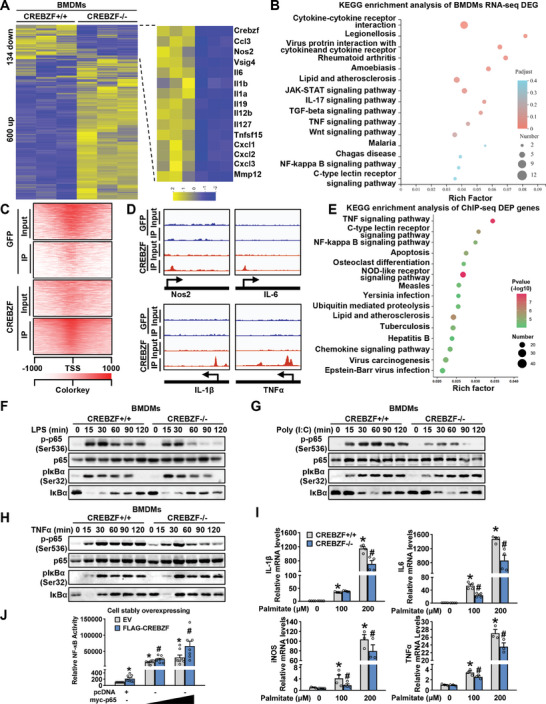
CREBZF deficiency in macrophages downregulated the NF‐κB pathway. A,B) BMDMs from CREBZF MKO and WT mice were stimulated with 100 ng mL^−1^ LPS for 6 h followed by RNA‐seq, *n* = 4. A) Heatmap representing global differentially expressed genes, along with the inflammation‐related genes. Shown 3 represent individuals of each group. B) KEGG enrichment analysis showed the most significantly enriched biological process. C–E) BMDMs from CREBZF MKO and WT mice were stimulated with 100 ng mL^−1^ LPS for 6 h followed by ChIP‐seq using myc antibody. C) Heatmaps of Input and myc‐CREBZF ChIP‐Seq signals at TSSs (±1 kb). D) Snapshot of the myc‐CREBZF ChIP‐Seq signal at inflammation‐related genes. E) KEGG enrichment analysis showed the most significantly enriched biological process. Immunoblot analysis in BMDMs from CREBZF MKO and WT mice treated 100 ng mL^−1^ LPS (F), 20 µg mL^−1^ poly (I:C) (G), and 20 ng mL^−1^ TNFα as indicated time points (H). I) Relative mRNA abundance of inflammatory genes in CREBZF^+/+^ and CREBZF^−/−^ BMDMs treated with palmitate for 24 h. *n* = 3–4. ^*^ *p* < 0.05 versus CREBZF^+/+^ BMDMs treated with vehicle; ^#^ *p* < 0.05 versus CREBZF^+/+^ BMDMs treated with palmitate as indicated. J) The NF‐κB‐luc activities in HEK293T cells stably overexpressing empty vector (EV) or CREBZF were analyzed by dual luciferase assay. *n* = 7. ^*^ *p* < 0.05 versus st.con and pcDNA; ^#^ *p* < 0.05 versus st.CREBZF and myc‐p65 as indicated.

To address the mechanisms that mediate the effects of CREBZF on macrophage functions, RNA sequencing of the WT and CREBZF deficient macrophages treated with LPS was performed. Of the annotated genes, 600 upregulated and 134 downregulated genes were identified, and CREBZF deficiency typically impaired inflammatory cytokines and chemokines gene expression (**Figure** **4**A). As expected, KEGG analysis revealed that the most prominent enrichment of altered biological system was associated with immune response (Figure 4B). Next, chromatin immunoprecipitation sequencing (ChIP‐seq) was performed to assess genes under the direct control of CREBZF according to its function as a transcriptional coregulator. In line with the roles of CREBZF in transcriptional regulation, annotated peaks in genomic positions revealed many peaks were positioned proximal to the transcription start site (TSS) (Figure 4C). Interestingly, several previously known proinflammation genes harbor CREBZF occupancies within the core transcription regions (Figure 4D). Notably, the nuclear factor kappa‐light‐chain‐enhancer of activated B cells (NF‐κB) signaling pathway appeared in KEGG analysis derived from both RNA‐seq and ChIP‐seq (Figure 4B,E). Given the pivotal roles of NF‐κB signaling in regulating multitudes of proinflammatory mediators, including cytokines, chemokines, and adhesion molecules,^[^ ^18^ ^]^ we explored whether CREBZF modulates NF‐κB activity.

NF‐κB signaling is activated by various stimuli,^[^ ^19^ ^]^ we investigated the potential function of CREBZF under the activation of TLR4 by LPS, TLR3 by polyinosinic‐polycytidylic acid (poly (I:C)), and TNFR by TNFα. The NF‐κB family comprises five transcription factors forming a homomeric or heterodimeric complex. Among the NF‐κB dimers, p65 (RelA) and p50 complex are predominant and typically sequestered in the cytoplasm mediated by its inhibitory proteins termed IκBα. Responding to stimulus, NF‐κB inhibitor IκBα is phosphorylated by IKK followed by degradation resulting in NF‐κB nuclear translocation and activation.^[^ ^20^ ^]^ As shown in Figure 4F–H, loss of CREBZF in macrophages reduced LPS‐, poly (I:C)‐ and TNFα‐induced phosphorylation status of p65, which is an essential event in nuclear NF‐κB signaling activation. Similarly, phosphorylation levels of p65 were also reduced in CREBZF‐deficient MEF cells treated with LPS or TNFα (Figure S7A–B, Supporting Information). CREBZF knockout efficiency was verified by real‐time PCR (Figure S7I, Supporting Information). Consistently, overexpression of CREBZF resulted in persistent phosphorylation of p65 in J774 cells (Figure S7C, Supporting Information). Previous studies demonstrated that nutritional fatty acids such as palmitic acid (PA) activated inflammatory signaling pathways,^[^ ^21^ ^]^ we further investigated whether CREBZF mediates the effects of PA‐induced activation of metabolic stress. Interestingly, CREBZF‐deficient BMDMs exhibited reduced expression of proinflammation genes in response to palmitate (Figure 4I). In addition, overexpression of CREBZF increased the NF‐κB luciferase activity (Figure 4J). Considering JNK signaling pathway regulates a multitude of proinflammatory mediators, we observed the similar activation of JNK and c‐Jun in WT and CREBZF deficient BMDMs under LPS treatment (Figure S7D, Supporting Information), indicating that CREBZF is not involved in JNK signaling pathway. Moreover, we observed similar degrees and kinetics of phosphorated IKK and phosphorated IκBα in CREBZF deficiency or overexpression cells compared with wild‐type cells (Figure 4F–H; Figure S7E–H, Supporting Information), indicating that CREBZF‐mediated upregulation of NF‐κB signaling was IKK‐independent. The results demonstrate that CREBZF is a positive regulator of NF‐κB signaling and likely functions downstream of IKK activation.

### 2.5 CREBZF Associates with p65 to Prevent Its Assembly with IκBα

**Figure 5:**
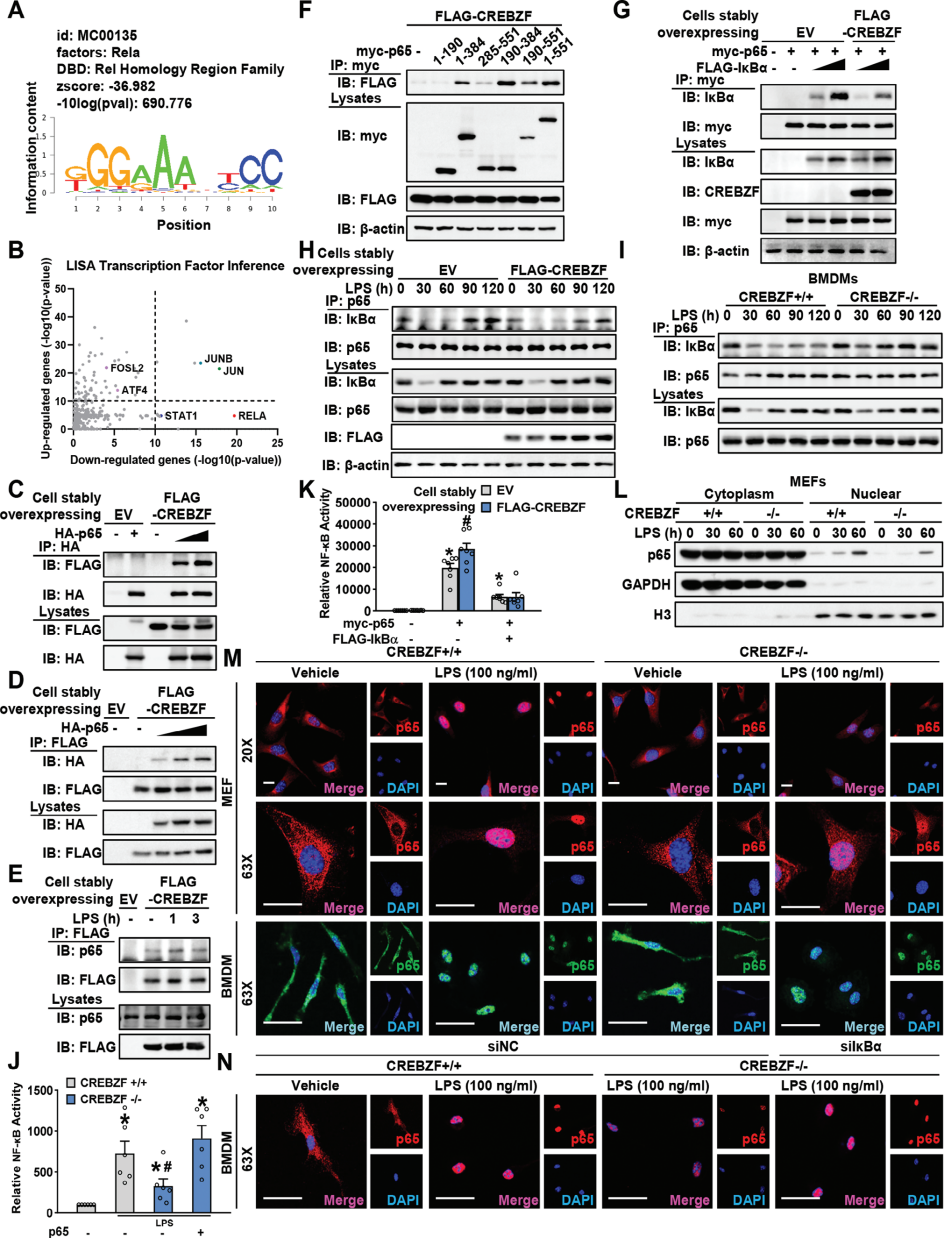
CREBZF extended the duration of nuclear p65 activity by inhibited the binding of IκBα to p65. A) Motif enrichment analysis of differential peaks of ChIP‐Seq. The DNA binding consensus sites of p65. B) LISA predicted transcription factors in regulating differential expression genes from RNA‐Seq. C,D) Immunoblot showing association between CREBZF and p65. HEK293T cells stably overexpressing empty vector (EV) or FLAG‐tagged CREBZF were transfected with HA‐tagged p65. The cell lysates were immunoprecipitated with HA antibody or FLAG antibody. E) J774 cells stably overexpressing empty vector (EV) or FLAG‐tagged CREBZF were treated with 100 ng mL^−1^ LPS. The cell lysates were immunoprecipitated with FLAG antibody. F) Mapping of interaction regions between CREBZF and p65. Myc‐tagged p65 and its truncate mutants were transfected within HEK293T cells stably overexpressing FLAG‐tagged CREBZF, followed by immunoprecipitation. G–I) CREBZF inhibited the interaction of p65 and IκBα. G) HEK293T cells stably overexpressing empty vector (EV) or FLAG‐tagged CREBZF were co‐transfected with myc‐tagged p65 and FLAG‐tagged IκBα followed immunoprecipitation via myc antibody. H) J774 cells stably overexpressing empty vector (EV) or FLAG‐tagged CREBZF were treated with 100 ng ml^−1^ LPS at indicated time points. The cell lysates were immunoprecipitated with p65 antibody. I) BMDMs from CREBZF MKO and WT mice were treated with 100 ng ml^−1^ LPS at indicated time points. The cell lysates were immunoprecipitated with a p65 antibody. J) CREBZF regulated NF‐κB activities via p65. The NF‐κB‐luc activities in CREBZF^+/+^ and CREBZF^−/−^ MEF transfected with pcDNA or p65 were analyzed by dual luciferase assay. MEF were treated with 100 ng ml^−1^ LPS. *n* = 6. ^*^ *p* < 0.05 versus CREBZF^+/+^ and pcDNA; ^#^ *p* < 0.05 versus CREBZF^+/+^ treated with LPS. K) CREBZF regulated p65 activities via IκBα. The NF‐κB‐luc activities in HEK293T cells stably overexpressing empty vector (EV) or FLAG‐tagged CREBZF were transfected with pcDNA, p65, or IκBα were analyzed by dual luciferase assay. *n* = 7. ^*^ *p* < 0.05 versus EV and pcDNA; ^#^ *p* < 0.05 versus EV and p65. L) The cytoplasm and nucleus were separated from LPS‐treated or ‐untreated CREBZF^+/+^ and CREBZF^−/−^ MEF to detect the amount of p65. M) Immunofluorescence of endogenous p65 and DAPI in CREBZF^+/+^ and CREBZF^−/−^ MEF (up), and CREBZF^+/+^ and CREBZF^−/−^ BMDMs (down) under 100 ng ml^−1^ LPS stimulation for 1 h. N) Immunofluorescence of endogenous p65 and DAPI in CREBZF^+/+^ and CREBZF^−/−^ BMDMs transfected with siNC or siIκBα. Scale bars, 20 µm.

Next, we explored molecular mechanisms of NF‐κB activation by CREBZF. We further analyzed the omics data mentioned in Figure 4. Annotated peaks of ChIP‐seq were performed comprehensive motif enrichment analysis and gave a list of predicted transcription factors (**Figure** **5**A; Figure S8A, Supporting Information). In parallel, we used a computational method called LISA^[^ ^22^ ^]^ to infer the likely transcription factors underlying the differentially expressed genes of RNA‐seq (Figure 5B). Interestingly, p65 (RelA) and JUNB were the top factors in both analyses (Figure 5B; Figure S8A, Supporting Information). In LISA analysis, JUNB was enriched both by the genes downregulated or upregulated upon CREBZF deficiency, but p65 was only enriched by the downregulated genes. The analysis gave us more clues to further explore the potential links between CREBZF and p65.

Given that CREBZF functions as a coregulator for transcription factors, we hypothesize that CREBZF may interact with p65. As shown in Figure 5C,D, CREBZF and p65 are associated with each other. Importantly, the increased interaction was induced by LPS treatment (Figure 5E). Next, the regions of p65 were mapped to find out the regions that are responsible for binding to CREBZF. The result showed that the 190–285 regions of p65 is sufficient to interact with CREBZF (Figure 5F; Figure S8B, Supporting Information). Interestingly, this region harboring containing the RHD domain is responsible for dimerization with IκBα which is the inhibitory protein of NF‐κB. The newly synthesized IκBα shows nucleocytoplasmic shuttling properties and retrieves nuclear NF‐κB complexes, contributing to the termination of NF‐κB transcription activity.^[^ ^23^ ^]^ Thus, we further hypothesized that the binding of CREBZF to p65 might affect its interaction with IκBα. As shown in Figure 5G, overexpression of CREBZF resulted in reduced interaction between IκBα and p65. Moreover, overexpression of CREBZF decreased endogenous newly synthesized IκBα bound to p65 (Figure 5H). Consistently, CREBZF deficiency caused an induction of binding between IκBα and p65 (Figure 5I). The interaction model based on the structure of the p65/p50 and the AlphaFold predicted structure of CREBZF was analyzed by protein–protein docking, indicating the similar spatial position of CREBZF and IκBα interacted with p65/p50 (Figure S8C, Supporting Information). The results support the notion that, following LPS treatment, CREBZF may bind to the dimerization domain of p65 and inhibit the interaction between p65 and IκBα, thus contributing to prolonged termination of p65 activity.

The reduction of NF‐κB luciferase activity mediated by CREBZF deficiency was abolished by p65 overexpression (Figure 5J), and overexpression of CREBZF enhanced NF‐κB luciferase activity which was blocked by IκBα treatment (Figure 5K). Finally, given the potential roles of CREBZF on the termination of p65, we tested whether CREBZF affected p65 nuclear content. As shown in Figure 5L,M, CREBZF deficiency decreased p65 nuclear location after LPS treated for 1 h. Moreover, the decreased nuclear p65 in CREBZF deficient cells was rescued by IκBα knockdown (Figure 5N). Taken together, these results indicate that CREBZF functions as a positive regulator of NF‐κB signaling via prolonging the duration of p65 activity by competing with IκBα.

### 2.6 Improved ATM Activation and Type 2 Diabetes by Pharmacological Inhibition of CREBZF in Insulin‐Resistant Mice

**Figure 6:**
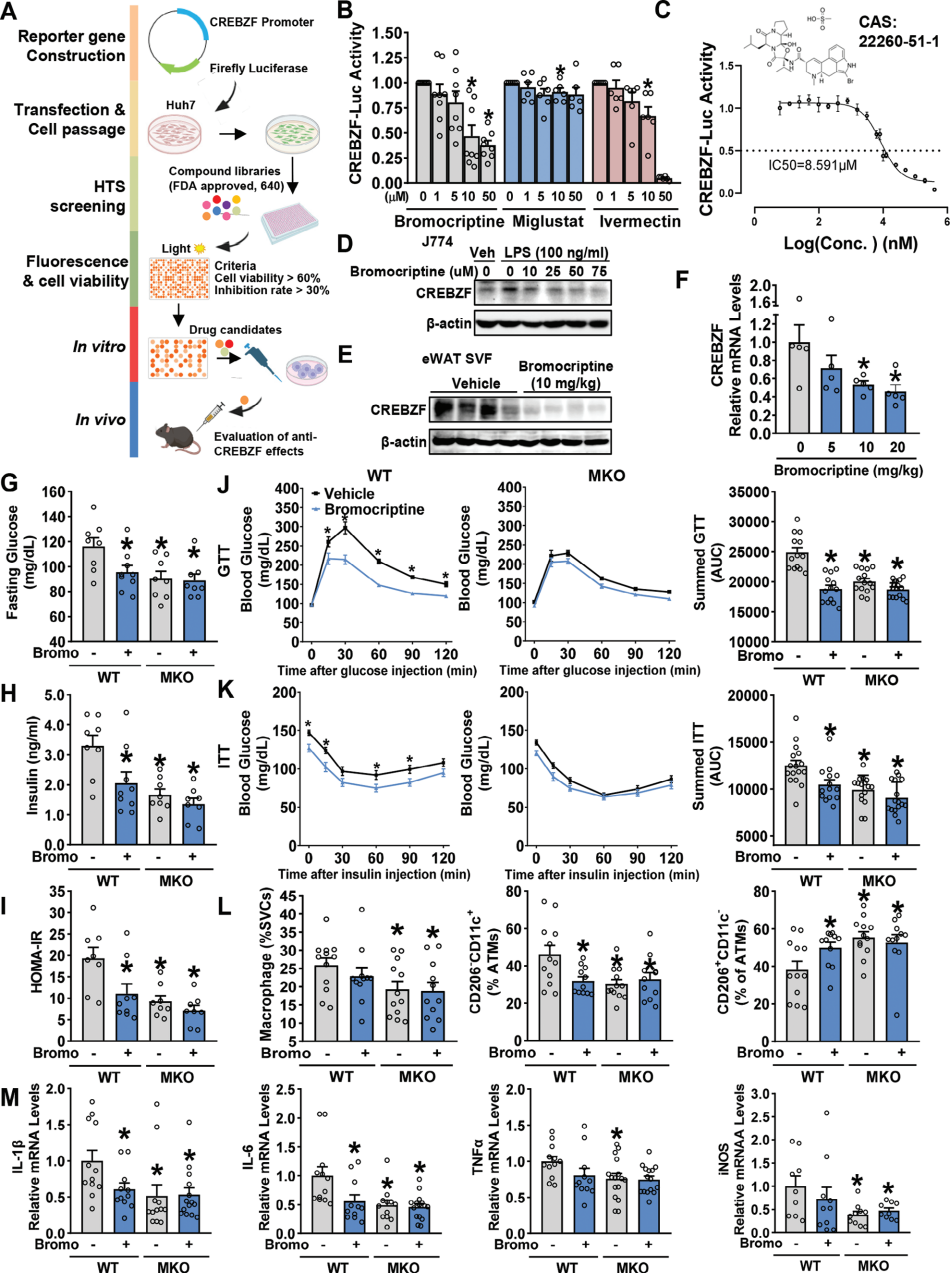
Pharmacological inhibition of CREBZF improved metabolic dysfunctions and reduced inflammation in HFHS diet‐induced obese mice. A) Schematic depicting the high‐throughput screening (HTS) strategy to identify CREBZF inhibitors. B) The inhibitory effects of compounds on CREBZF were assessed in stably transfected CREBZF‐promoter‐luciferase Huh7 cells. Cells were incubated with different concentrations as indicated for 48 h. C) The inhibitory curve of bromocriptine mesylate. D) J774 cells were incubated with bromocriptine mesylate for 48 h and then treated with 100 ng mL^−1^ LPS for 4 h. E) Immunoblot showing CREBZF protein levels in SVF from eWAT with or without bromocriptine mesylate injection. C57BL/6N mice were intraperitoneally injected with 10 mg kg^−1^ bromocriptine mesylate daily for 1 week. F) Relative mRNA abundance of CREBZF in SVF from eWAT with or without bromocriptine mesylate injection. G–M) C57BL/6N mice were intraperitoneally injected with 5, 10, or 20 mg kg^−1^ bromocriptine mesylate daily for 1 week. Male CREBZF MKO and WT mice at 8 weeks old were fed with HFHS diet for 12 weeks followed by intraperitoneal injection with vehicle or 10 mg kg^−1^ bromocriptine mesylate daily for 4 weeks. G) Fasting blood glucose and H) plasma insulin levels were assessed. I) HOMA‐IR. J) The glucose tolerance test (1 g kg^−1^) and respective area under the curve were performed. K) Insulin tolerance test (1 U kg^−1^) and respective area under the curve were performed. G–I) *n* = 8–9. ^*^ *p* < 0.05 versus WT mice and vehicle. J–K) *n* = 13–14. ^*^ *p* < 0.05 versus WT mice and vehicle. L) Quantification of the F4/80^+^CD11b^+^ macrophages of eWAT SVF, the CD11c^+^CD206^−^ and CD11c^−^CD206^+^ subsets of total macrophages from eWAT. *n* = 8–9. ^*^p < 0.05 versus WT mice and vehicle. M) Relative mRNA abundance of inflammatory genes of eWAT SVF. *n* = 10–14. ^*^ *p* < 0.05 versus WT mice and vehicle.

To test the efficacies of pharmacological inhibition of CREBZF in the attenuation of obesity‐associated inflammation and insulin resistance, we adapted a luciferase assay to a high‐throughput screening (HTS) strategy for identifying potential compounds that repress the transcription of CREBZF (**Figure** **6**A). Firefly luciferase was widely used as a reporter to monitor the activity of a specific target under chemical stimulus. The effects of 640 compounds from the FDA‐approved library on CREBZF transcriptional activity were evaluated through the high‐throughput screening system in stable cell lines expressing firefly luciferase under the control of CREBZF promoter (Figure S9A, Supporting Information).In parallel, PrestoBlue dye assay was utilized to assess the cytotoxicity of the compounds, and normalization of firefly luciferase activity with PrestoBlue dye assay assisted in compensating for potential variability in cell density and cell viability. Afterward, 70 compounds were selected as hits, which were performed HTS again (Figure S9B–D, Supporting Information). The top three candidates including bromocriptine mesylate (Bromo), miglustat, and ivermectin were evaluated as potential CREBZF inhibitors, and we analyzed their dose‐dependent inhibitory effects (Figure 6B). Although ivermectin showed dramatic inhibition on CREBZF at 50 µm, violent cell cytotoxicity was observed (data not shown). Therefore, we identified bromocriptine mesylate as the most potential CREBZF inhibitor with an overall assessment including inhibition efficiency, and cell cytotoxicity and measured the inhibition curve (Figure 6C). Although the utility of bromocriptine in illness is mediated by its dopamine agonist activity on dopamine receptor D2 (DRD2),^[^ ^24^ ^]^ we found that the inhibitory effects of bromocriptine on CREBZF are independent on DRD2 (Figure S9E–G, Supporting Information). Interestingly, we found bromocriptine mesylate reduced CREBZF stability in cells stably overexpressing FLAG‐tagged CREBZF (Figure S9H, Supporting Information). Bromocriptine mesylate also inhibited LPS‐induced CREBZF (Figure 6D). Further studies revealed that bromocriptine mesylate suppresses CREBZF not only in vitro but also in vivo (Figure 6E,F). Together, these results demonstrate that bromocriptine functions as a CREBZF inhibitor.

We further evaluated the therapeutic effects of bromocriptine on diet‐induced obese mice. CREBZF MKO mice and their WT littermates were fed on an HFHS diet for 12 weeks, followed by intraperitoneal injection with bromocriptine mesylate for 4 weeks, or vehicle as a control. In WT mice, treatment with bromocriptine mesylate significantly improved glucose tolerance and insulin resistance (Figure 6G–K). The reduction of macrophage content and the proportion of proinflammatory macrophages in eWAT were observed (Figure 6L). Consistently, proinflammation genes were decreased in SVF isolated of eWAT from mice injected with bromocriptine mesylate (Figure 6M). Strikingly, the beneficial effects of bromocriptine mesylate were abolished in CREBZF MKO mice. These results demonstrate that macrophage CREBZF is required for the beneficial effects of bromocriptine mesylate on the attenuation of ATM activation, insulin resistance, and type 2 diabetes in mice, and CREBZF may serve as a therapeutic target to relieve a ATM proinflammatory activation in insulin resistance.

### 2.7 High CREBZF Levels in ATM Are Correlated with Adipose Inflammation and Insulin Resistance in Obese Patients

**Figure 7:**
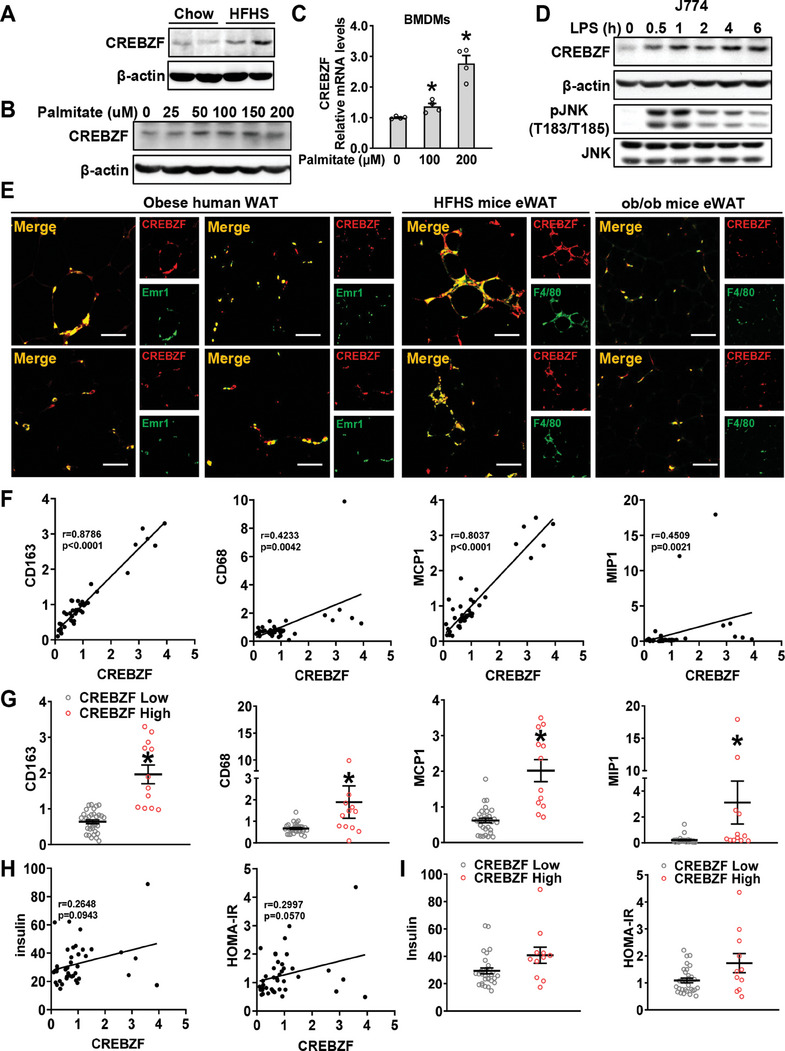
CREBZF correlated with adipose inflammation in obese patients. A) Protein levels of CREBZF in SVF of eWAT from C57BL/6N mice fed a chow or HFD diet for 16 weeks. B) BMDMs were incubated with 0, 25, 50, 100, 150, 200 µM palmitate for 24 h. C) Relative CREBZF mRNA levels in BMDMs treated with 0, 100, and 200 µM palmitate for 24 h. D) Protein levels of CREBZF in J774 cells treated with 100 nm LPS at indicated time. *n* = 4. E) Representative CREBZF and F4/80 double immunofluorescence of VAT from obese patients, eWAT from C57BL/6N mice fed HFHS diet for 16 weeks, eWAT from ob/ob mice. Scale bars, 50 µm. F) Correlation between transcriptional levels of CREBZF with CD163, CD68, MCP‐1, and MIP‐1 in VAT from obese individuals. *n* = 44. G) Relative CD163, CD68, MCP‐1, and MIP‐1 mRNA levels in high expression CREBZF group and low expression group. *n* = 44. ^*^ *p* < 0.05 versus CREBZF low group. H) Correlation between transcriptional levels of CREBZF and insulin and HOMA‐IR in obese patients. *n* = 41. I) Insulin levels and HOMA‐IR in high expression CREBZF group and low expression group. *n* = 41. Data are presented as means ± SEM, *p* values are determined by unpaired two‐tailed Student's *t*‐test. Spearman's correlation analysis shows *R* values, and two‐tailed *p* values (F,H).

We observed CREBZF expression was increased in stromal vascular fraction cells (SVF) of diet‐induced obese mice, where macrophages are typically present (**Figure** **7**A). To investigate whether CREBZF is responsive to obesity‐associated proinflammatory stimuli and insulin signals, the macrophages were treated with palmitate or LPS. Interestingly, we found CREBZF was induced in macrophages in a dose‐dependent or time‐dependent manner (Figure 7B–D). Notably, the expression levels of CREBZF were not changed by insulin (Figure S10A,B, Supporting Information), although insulin induces CREBZF expression in hepatocytes.^[^ ^8^ ^]^ Consistently, double immunofluorescence of CREBZF and macrophage marker F4/80 showed CREBZF colocalized with F4/80 in the crown‐like structures of adipose sections from obese human and mice, suggesting that CREBZF is highly expressed in macrophages (Figure 7E).

We also found a significant positive correlation between CREBZF and transcript levels of proinflammatory genes, such as monocyte/macrophage lineage markers CD163 and CD68, and the proinflammatory proteins like MCP‐1 and MIP‐1 in overweight/obese subjects (Figure 7F; Table S1, Supporting Information). When the subjects were classified into CREBZF high and CREBZF low groups according to average expression levels of CREBZF, the CREBZF high subjects exhibited more severe inflammation (Figure 7G). Among the clinical quantitative traits, fasting insulin levels and HOMA‐IR were the most relevant metabolic characteristics correlated with CREBZF transcript levels, although there was no difference (Figure 7H,I). In parallel, we analyzed a transcriptome data set, including 434 human subcutaneous adipose tissues from the Gene Expression Omnibus (GEO) database.^[^ ^25^ ^]^ Consistently, CREBZF expression was positively correlated with proinflammatory cytokines and myeloid lineage markers significantly (Figure S10C,D, Supporting Information). Given the link between inflammation and lipid metabolism in adipose tissue, we further analyzed lipid metabolic genes. Notably, CREBZF was positively correlated with genes regulating lipid synthesis and lipolysis but negatively correlated with lipid droplet protein transcript levels (Figure S10E–G, Supporting Information). Taken together, the in vivo evidence suggests that CREBZF is required for the beneficial effects of bromocriptine on anti‐inflammatory phenotype and improved insulin sensitivity in mice, and pharmacological inhibition of CREBZF may have potential therapeutic effects on metabolic disorders.

## 3 Discussion

This study demonstrates that CREBZF acts as a novel regulator in macrophage‐mediated inflammation and insulin resistance. Additionally, the findings of CREBZF on regulating NF‐κB signaling represent an unknown mechanism that underlies chronic low‐grade inflammation associated with overnutrition. The interaction between CREBZF and p65 inhibits IκBα binding to p65, resulting in increased nuclear localization of p65 and enhanced NF‐κB activity which enhances transcription of proinflammatory gene expression. Moreover, we demonstrate that pharmacological inhibition of CREBZF attenuates inflammation and insulin resistance in obese mice (Figure S11, Supporting Information).

### 3.1 Overnutrition‐Induced Transcriptional Coregulator Couples ATM Activation to Insulin Resistance

This current study identifies that overnutrition and inflammation increase the expression of CREBZF. First, CREBZF is highly expressed in ATM of obese humans, and human studies reveal a significantly positive correlation between expression levels of CREBZF and several pro‐inflammatory genes. Second, CREBZF in ATM is upregulated in mice under HFHS diet feeding. Furthermore, obesity‐associated proinflammatory stimuli like palmitic acid or LPS induced CREBZF in macrophages. The findings broaden our points of CREBZF responding to different pathological conditions.

This study characterizes a previously unknown function for CREBZF in regulating macrophage‐mediated inflammation and insulin resistance. First, myeloid CREBZF deficiency profoundly reduced chronic inflammation and metabolic dysfunctions in HFHS diet‐induced diabetic mice. Moreover, CREBZF deficiency decreased the proinflammatory activities of ATM, which preceded the improvement of hyperglycemia and insulin resistance. Notably, specific depletion of neutrophils did not abolish the augmentative effects of myeloid CREBZF deficiency on attenuating insulin resistance. Collectively, these findings demonstrate that CREBZF is dynamically regulated in response to inflammatory stimuli, and hyper‐activation of CREBZF in macrophages contributes to systemic insulin resistance and type 2 diabetes. Given that hepatic macrophages have been implicated in the process of NAFLD and NASH, the effects of macrophage CREBZF on these disorders require further investigation.

### 3.2 CREBZF Serves as a Positive Regulator of p65

NF‐κB signaling is known as an essential transcriptional mechanism of macrophage activation and a master regulator linking inflammation and insulin resistance.^[^ ^18^, ^26^ ^]^ However, the precise signaling cascades in NF‐κB signaling remain obscure, remarkably the mechanism of hyperactive NF‐κB under overnutrition is largely unknown. Our findings show that CREBZF functions as a coregulator to promote NF‐κB transcriptional activity, which underlies chronic low‐grade inflammation associated with overnutrition. By interacting with the dimerization part of p65 in its RHD domain, CREBZF inhibits the binding of IκBα to p65, thus enhancing p65 nuclear localization and transcriptional activity. This model is supported by several evidences. First, CREBZF gain‐ and loss‐of‐function approaches demonstrate that CREBZF controls the induction of several NF‐kB target genes. Second, the immunoprecipitation assays confirm the association between CREBZF and p65. Third, the competitive binding assays verify that CREBZF inhibits the association of p65 with IκBα, which prolongs the termination and duration of nuclear p65. Therefore, CREBZF shows biological significance in regulating NF‐κB signaling.

Although the termination of NF‐κB activity relying mainly on the resynthesis of IκB proteins is generally accepted,^[^ ^23^ ^]^ the precise regulation is still largely unknown, especially related to various pathological and physiological processes.^[^ ^27^ ^]^ Recent studies have described p300/HDAC,^[^ ^28^, ^29^ ^]^ and Pin1^[^ ^30^ ^]^ alter IκBα binding through post‐translational modification of p65. Our report characterizes CREBZF act as a NF‐κB coregulator in the nucleus, disrupting the conventional negative feedback loop mediated by IκBα. Notably, moderate NF‐κB activity is essential in monocytes undergoing macrophage and myeloid dendritic differentiation.^[^ ^31^, ^32^ ^]^ Given that myeloid CREBZF deficiency does not participate in immune cell differentiation, it indicates that the CREBZF‐NF‐κB axis functions under specific physiological circumstances. Moreover, CREBZF is increased in macrophages of mice fed with a HFHS diet, palmitate, and LPS elicit a robust induction of CREBZF in BMDMs, which may underscore the specific role of CREBZF under nutrient overload conditions. These findings support a finely tuned cellular mechanism in regulating the duration of nuclear NF‐κB action.

### 3.3 Inhibition of CREBZF Improves Insulin Sensitivity via the Crosstalk Between ATM and Adipocytes

The current findings indicate chronic inflammation is an essential component of obesity‐related disorders, including insulin resistance.^[^ ^33^, ^34^ ^]^ The major challenge is finding causal targets of the disease. One of the most important findings is the therapeutic potential of targeting CREBZF in type 2 diabetes (T2DM). CREBZF‐deficient BMDMs show decreased proinflammatory activities and chemotactic capacities. The cocultured assays reveal that CREBZF deficiency in macrophages improves insulin sensitivity in primary adipocytes and adipose tissues. Given the clues that CREBZF regulates insulin resistance via the crosstalk between ATM and adipocytes, we screened a small molecule compound bromocriptine mesylate, which inhibits CREBZF to suppress inflammation and ameliorates insulin resistance.

Bromocriptine is a dopamine D2 receptor agonist suppressing the production of prolactin and was originally used for treating hyperprolactinemia, Parkinson's disease, and so on.^[^ ^24^ ^]^ Interestingly, bromocriptine was approved by the FDA for treating T2DM in 2009.^[^ ^35^ ^]^ With the present data, the therapeutic effects of bromocriptine on hyperglycemia are independent of the known mechanisms, such as prolactin, MC4R, or circadian rhythms.^[^ ^36^ ^]^ The precise mechanisms underlying the antidiabetic effects of bromocriptine remain unclear. In addition, bromocriptine treatment shows reduced low‐grade systemic inflammation in clinical trials or preclinical studies,^[^ ^37^, ^38^ ^]^ but the underlying mechanisms are largely unknown. Our findings support CREBZF acts as a new mechanism of bromocriptine treatment. We identified bromocriptine in a blind screen for CREBZF inhibitors and showed that it partially prevents obesity‐related inflammatory and metabolic disorders by inhibiting CREBZF. Furthermore, pharmacological and genetic approaches for the modulation of CREBZF activity indicate it has the potential to be a therapeutic drug target.

Anti‐inflammation shows the therapeutic potential of T2DM.^[^ ^39^, ^40^ ^]^ Although most anti‐inflammatory agents in clinical trials have been proven negative, including anti‐IL‐1β or anti‐TNFα antibodies,^[^ ^41^ ^]^ salicylate or other salicylic acid derivatives reduce blood glucose in clinical trials by inhibiting IKK/NF‐κB,^[^ ^42^ ^]^ indicating therapeutic strategies able to modulate widely inflammatory agents show more potential than target a single cytokine. Our findings showing CREBZF as a NF‐κB coregulator under overnutrition, coupling with the effects of CREBZF on exacerbated hepatic steatosis and NASH, suggest that targeting CREBZF may represent general benefits in treating metabolic diseases. Notably, there are clear benefits to repurposing existing drugs to tackle different diseases. One promising approach to developing new uses for old drugs is finding new targets. Due to the inhibitory effects of bromocriptine on CREBZF, biological functions of CREBZF may give clues to expand indications of bromocriptine in various diseases such as NAFLD and NASH.

In summary, the current study demonstrates a novel mechanism of the CREBZF‐NF‐κB axis in the macrophage regulating inflammation and insulin resistance. CREBZF is induced by the overnutrition status in humans and mice, and the pharmacological and genetic inhibition of CREBZF attenuates symptoms of T2DM further solidifying the role of CREBZF in regulating hyperglycemia. This study suggests therapeutic possibilities for targeting CREBZF in T2DM.

## 4 Experimental Section

### Animal Model and Diets

Myeloid‐specific CREBZF knockout (CREBZF MKO) mice were generated by crossing floxed CREBZF mice containing the deleted open reading frame of CREBZF (Cyagen Biosciences, China) with LysM‐Cre mice in which the lysozyme promoter drives Cre.^[^ ^43^ ^]^ Wild‐type (WT) littermates (LysM‐Cre negative, CREBZF flox/flox) were used as the control. Mice were fed with a high‐fat, high‐sucrose (HFHS) diet (D12327; Research Diets) for 16 weeks. All mice were housed under a 12:12‐h light/dark cycle at a controlled temperature. All animal experimental protocols were approved by the Institutional Animal Care and Use Committee at the Shanghai Institute of Nutrition and Health, Chinese Academy of Sciences.

### Adipose Specimens from Humans

Visceral white adipose samples were obtained from adult patients undergoing bariatric surgery. Patients gave written consent for their samples to be collected. The study protocol was approved by the Ethics Committee of Nanjing Drum Tower Hospital, The Affiliated Hospital of Nanjing University Medical School, and was conducted in accordance with the 1975 Declaration of Helsinki.

### Isolation and Differentiation of Bone Marrow‐Derived Macrophages

BMDMs from 8‐ to 12‐week‐old mice were obtained as previously described.^[^ ^44^ ^]^ In brief, the femurs and tibiae were collected and flushed using a syringe with PBS. After filtrated and centrifuged, bone marrow cells were cultured in RPMI supplemented with 10% FBS and 10 ng mL^−1^ M‐CSF (Peprotech, 315‐02) for 7 days and allowed to differentiate into mature macrophages.

### Statistical Analysis

For clinical data, normally distributed data were expressed as mean ± SEM, and non‐normally distributed data were expressed as median with interquartile range. The Gaussian distribution was tested using the D'Agostino and Pearson omnibus normality test. Human correlations were performed using Spearman's test. Statistical significance between CREBZF levels and insulin was evaluated by the Mann–Whitney test. For animal and in vitro studies, data were expressed as mean±SEM. Statistical significance was evaluated by unpaired two‐tailed Student's *t*‐test to compare two groups. Statistical analyses were performed using statistical tools in GraphPad Prism 8.0. Differences were defined as significant at a *p* value < 0.05.

## Conflict of Interest

The authors declare no conflict of interest.

## Author Contributions

Y.L., W.S., and Z.L. contributed equally to this work. Y.L., W.S., Z.L., and Y.L. contributed to the experiment design; Y.L., W.S., Z.L., Z.H., J.S., Z.Z., D.D., W.H., W.L., G.C., S.W., N.L., and X.F. contributed to the acquisition and analysis of data; Y.B. provided reagents and material support; H.L., J.Q., H.Z., Y.X., and C.Z. edited the manuscript with important intellectual content; Y.L., A.C., and C.Z. obtained the funding; Y.L., W.S., A.C., C.Z., and Y.L. wrote the manuscript.

## Supporting information

Supporting Information

## Data Availability

The data that support the findings of this study are available in the supplementary material of this article.
