# Supplementary material for: Macrophage CREBZF Orchestrates Inflammatory Response to Potentiate Insulin Resistance and Type 2 Diabetes
Source: Adv Sci (Weinh). 2024 Jan 29;11(13):2306685. doi: 10.1002/advs.202306685 (PMC10987118; doi:10.1002/advs.202306685)
Supplement: Supplementary file 1 — Supporting Information [file ADVS-11-2306685-s001.pdf]

ADVANCED SCIENCE
Open Access

Supporting Information

for *Adv. Sci.*, DOI 10.1002/advs.202306685

Macrophage CREBZF Orchestrates Inflammatory Response to Potentiate Insulin Resistance and Type 2 Diabetes

Yuxiao Liu, Weitong Su, Zhengshuai Liu, Zhimin Hu, Jiaxin Shen, Zengpeng Zheng, Dong Ding, Wei Huang, Wenjing Li, Genxiang Cai, Shuang Wei, Ni Li, Xia Fang, Hong Li, Jun Qin, Haibing Zhang, Yichuan Xiao, Yan Bi, Aoyuan Cui, Chunxiang Zhang* and Yu Li**

Macrophage CREBZF orchestrates inflammatory response to potentiate insulin resistance and type 2 diabetes

Yuxiao Liu[1,7], Weitong Su[1,7], Zhengshuai Liu[1,7], Zhimin Hu[1], Jiaxin Shen[1], Zengpeng Zheng[1], Dong Ding[1], Wei Huang[2], Wenjing Li[1], Genxiang Cai[1], Shuang Wei[1], Ni Li[3], Xia Fang[1,2], Hong Li[4], Jun Qin[3], Haibing Zhang[1], Yichuan Xiao[3], Yan Bi[5], Aoyuan Cui[1]*, Chunxiang Zhang[6]*,Yu Li[1]*

[1]CAS Key Laboratory of Nutrition, Metabolism and Food Safety, Shanghai Institute of Nutrition and Health, University of Chinese Academy of Sciences, Chinese Academy of Sciences, Shanghai 200031, China; [2]Department of Endocrinology and Metabolism, The Affiliated Hospital of Southwest Medical University, Metabolic Vascular Diseases Key Laboratory of Sichuan Province, Luzhou, Sichuan 646000, China; [3]CAS Key Laboratory of Tissue Microenvironment and Tumor, Shanghai Institute of Nutrition and Health, University of Chinese Academy of Sciences, Chinese Academy of Sciences, Shanghai 200031, China; [4]CAS Key Laboratory of Computational Biology, Shanghai Institute of Nutrition and Health, Chinese Academy of Sciences, Shanghai 200031, China; [5]Affiliated Drum Tower Hospital, Medical School of Nanjing University, Nanjing, Jiangsu, 210008, China; [6]Metabolic Vascular Disease Key Laboratory of Sichuan Province, The Affiliated Hospital of Southwest Medical University, Key Laboratory of Medical Electrophysiology, Ministry of Education, Southwest Medical University, Luzhou, 646000, China. [7]These authors contribute equally to this work.

Running Title: Macrophage CREBZF potentiates insulin resistance

Supplementary Fig. S1 **Liu Y, et al.**

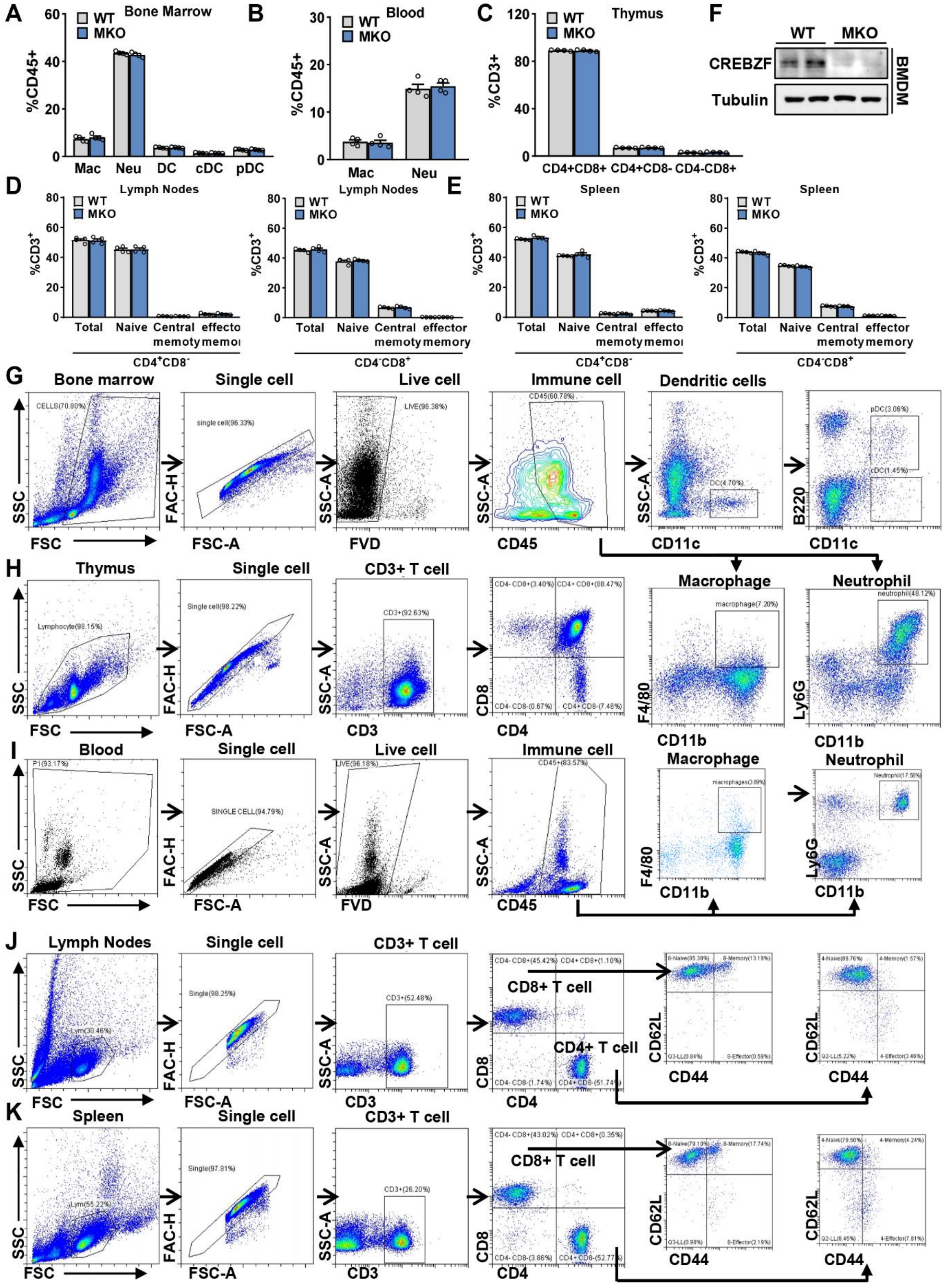

Supplementary Fig. S2

Liu Y, et al.

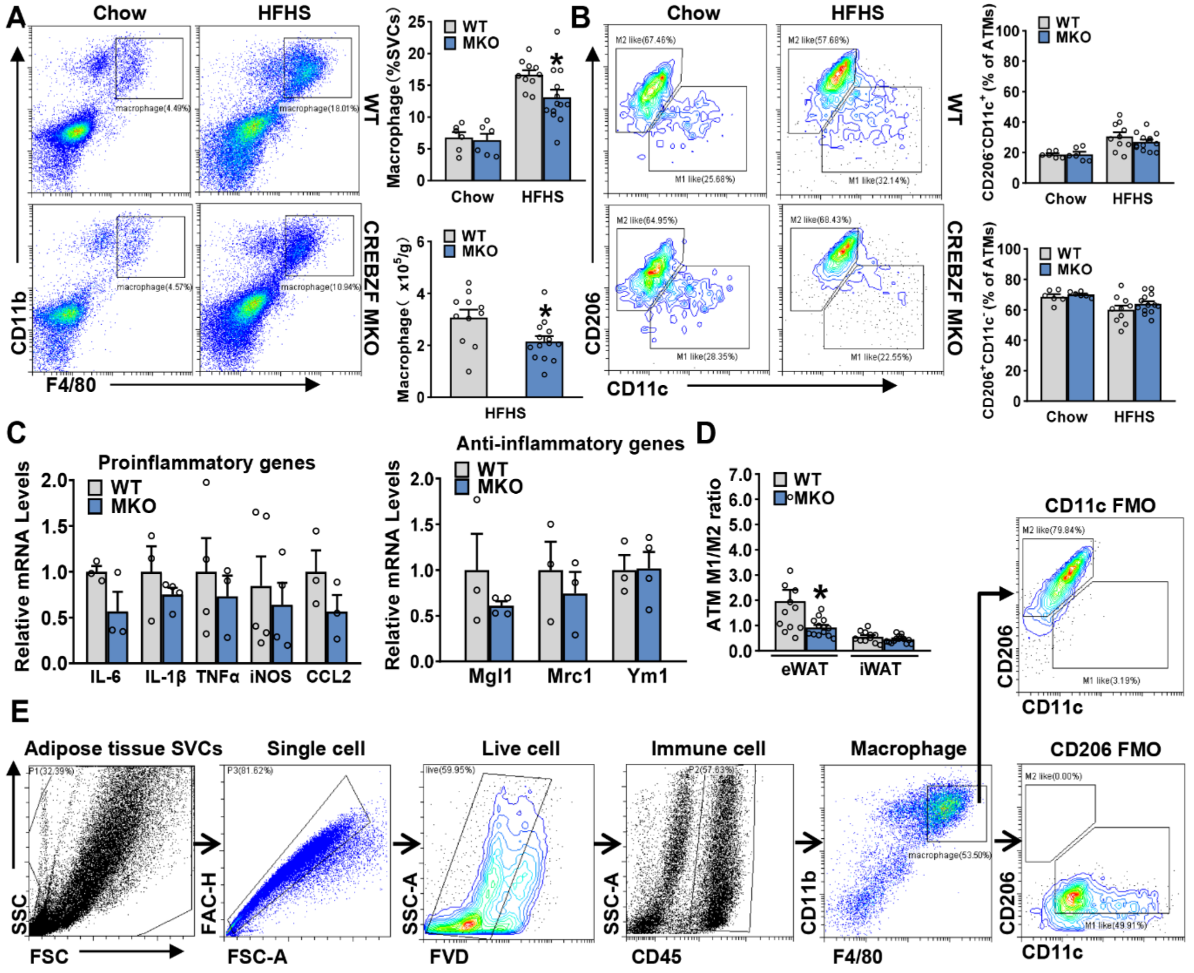

Supplementary Fig. S3

Liu Y, et al.

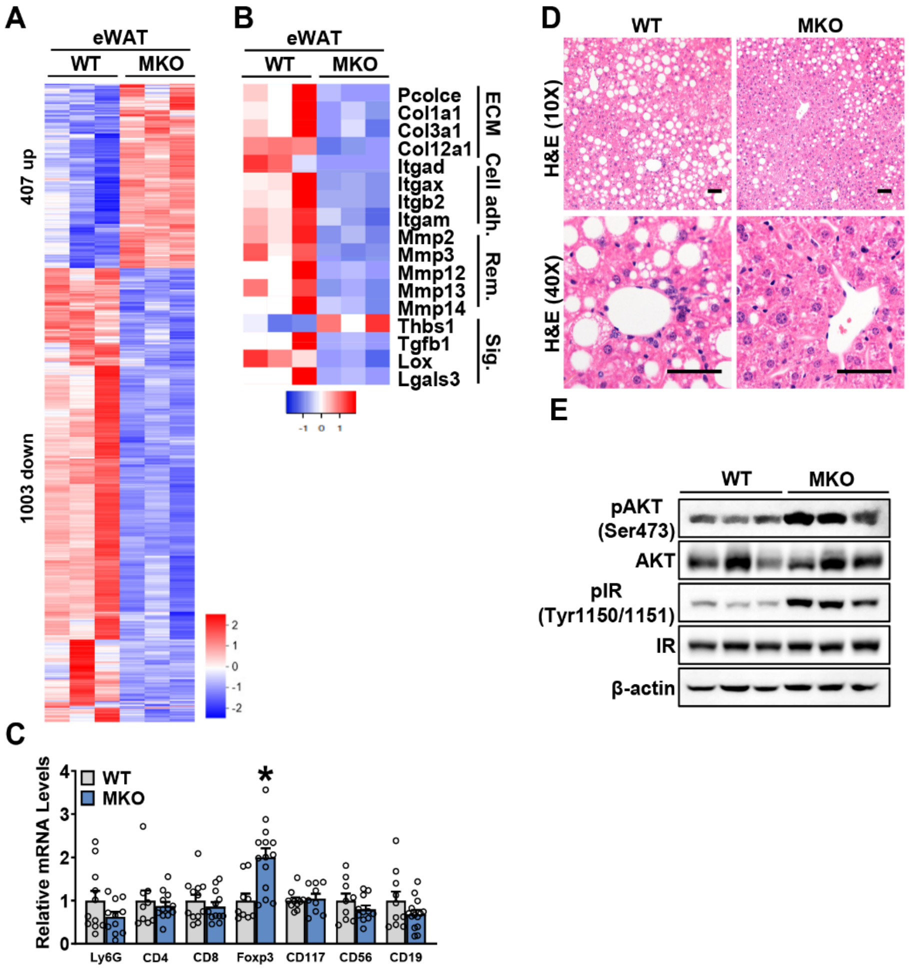

Supplementary Fig. S4

Liu Y, et al.

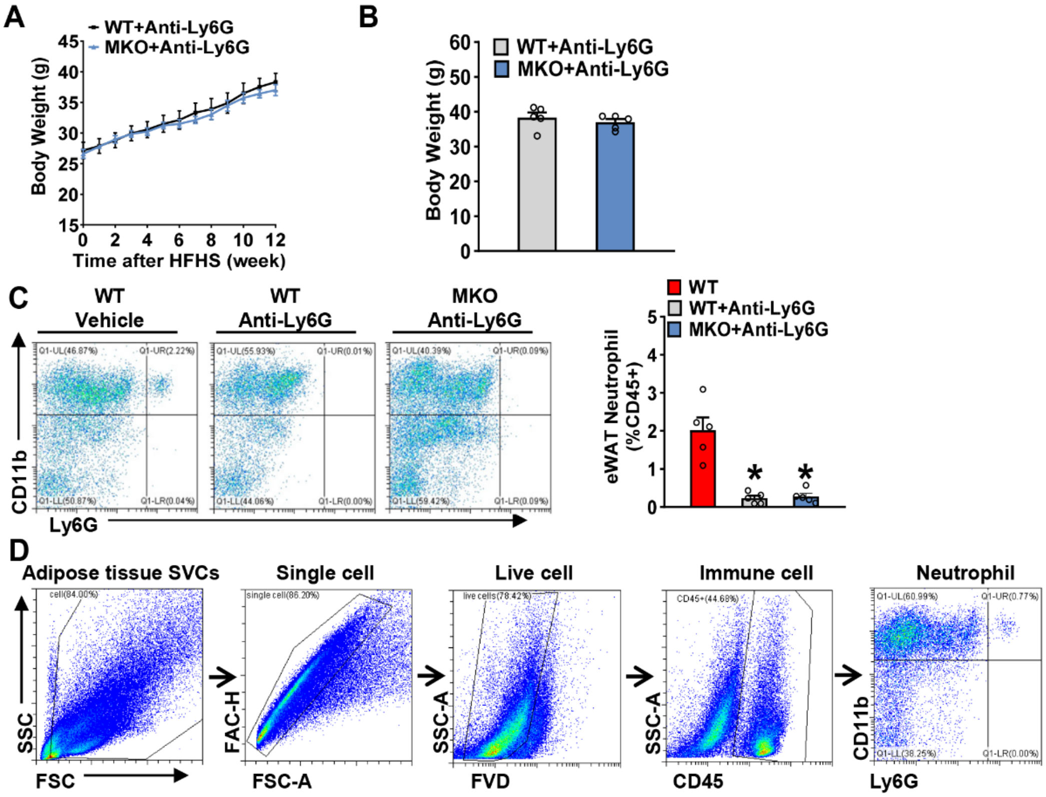

Supplementary Fig. S5

Liu Y, et al.

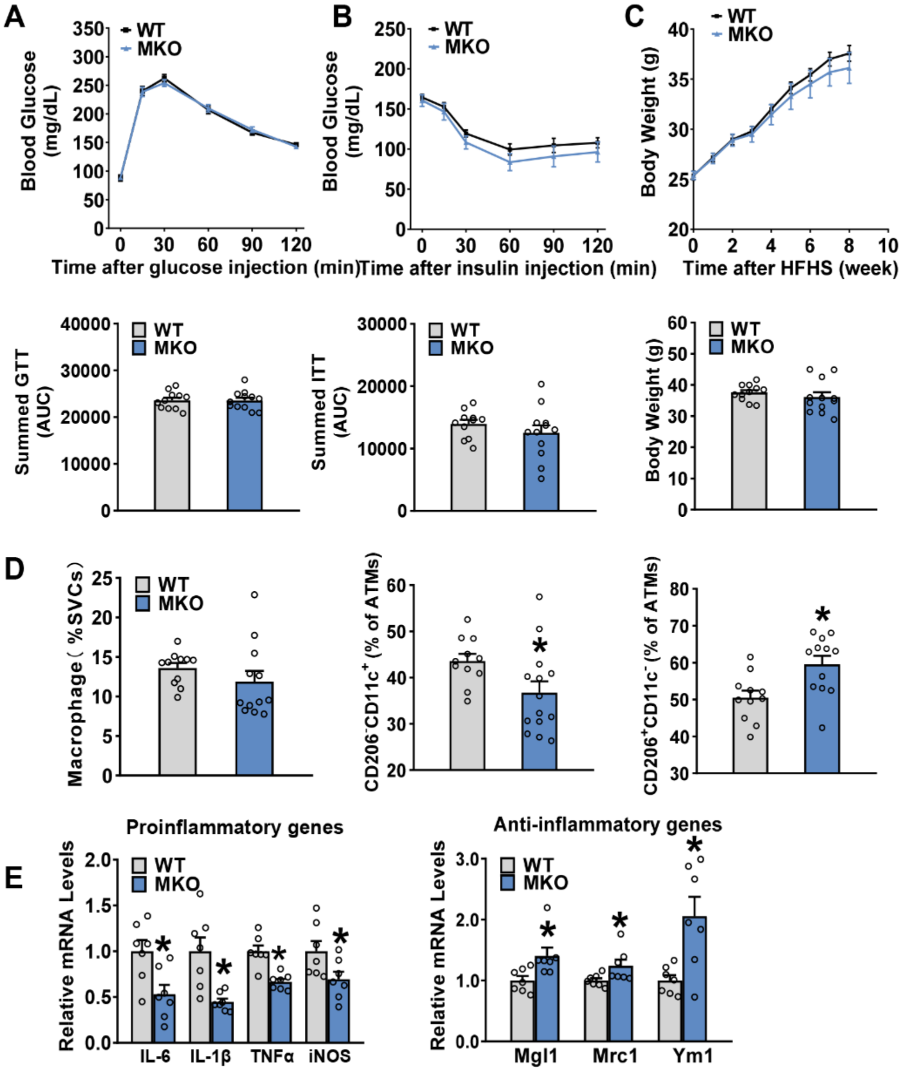

Supplementary Fig. S6

Liu Y, et al.

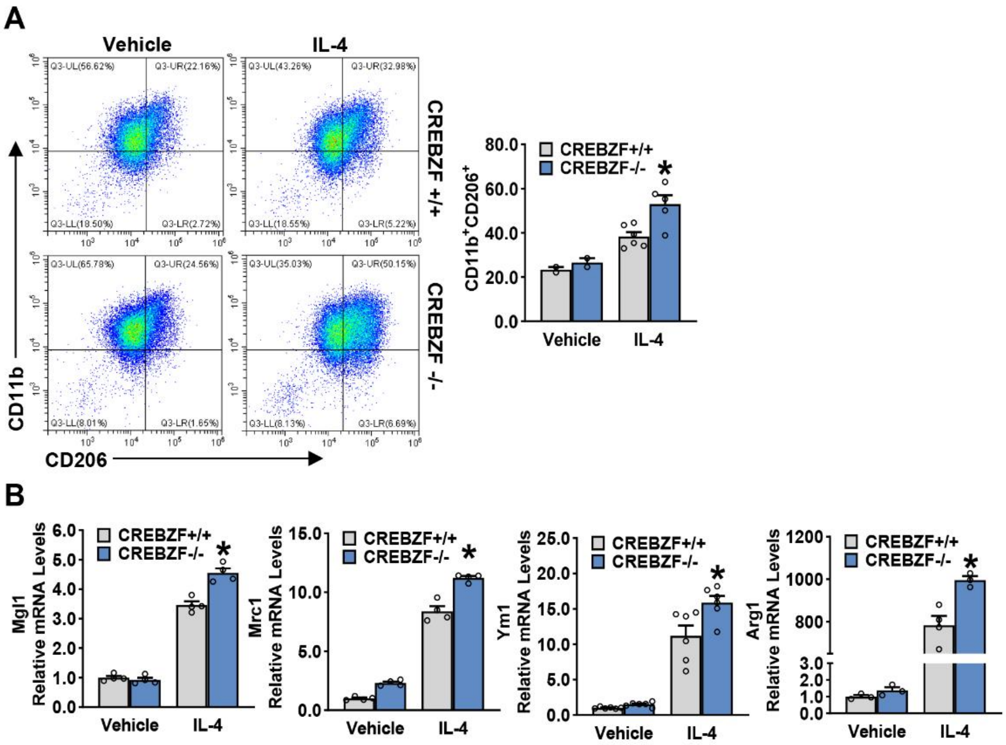

Supplementary Fig. S7

Liu Y, et al.

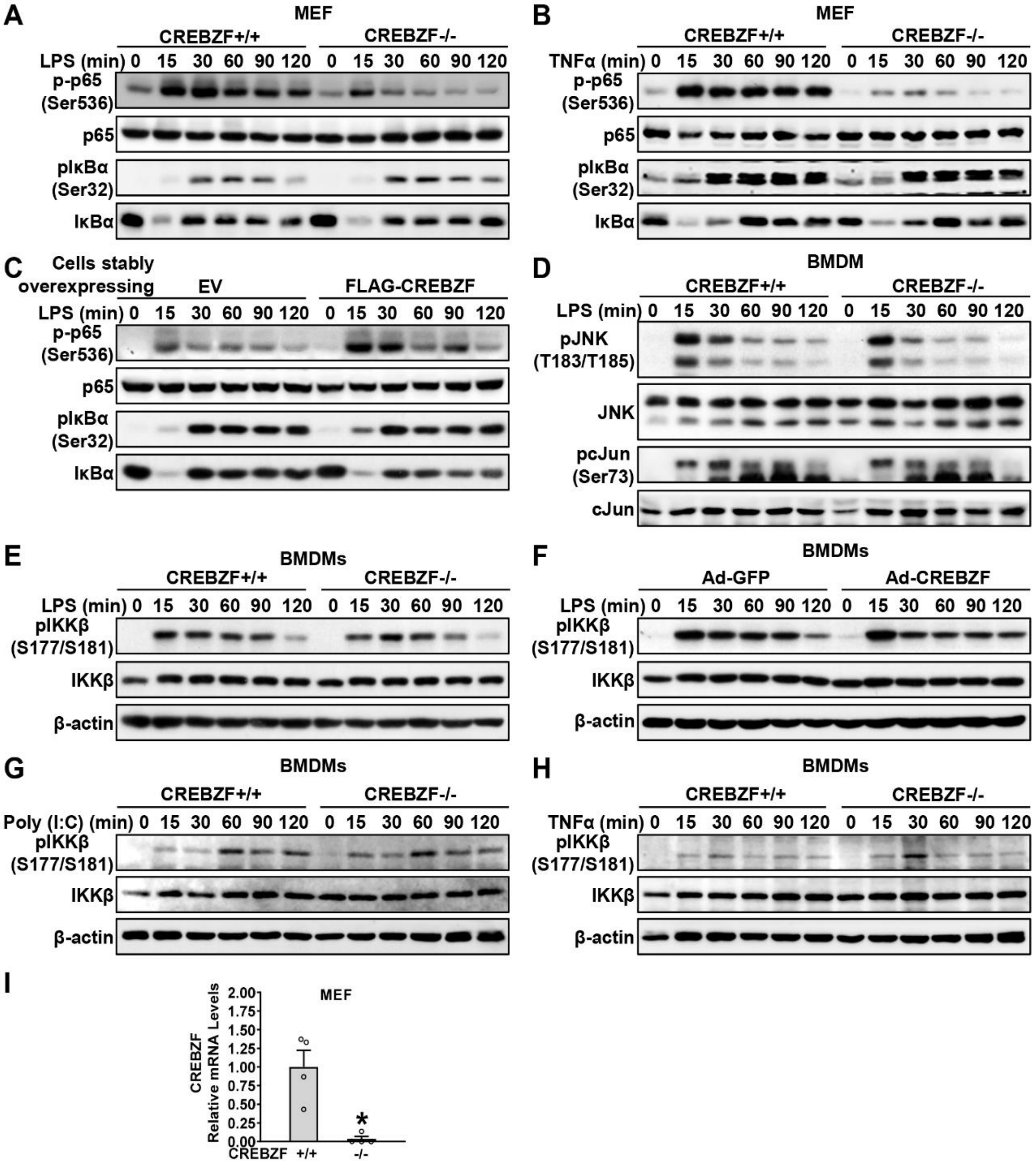

Supplementary Fig. S8

Liu Y, et al.

A

Factor	DNA binding domain	zscore
Rela	Rel Homology Region Family	-36.982
Sfpi1	Ets Domain Family	-31.073
ETV6	Ets Domain Family	-28.625
NFKB2	Rel Homology Region Family	-26.935
ELF5	Ets Domain Family	-26.516
JUNB	Leucine Zipper Family	-26.166
Cebpb	Leucine Zipper Family	-25.292

B

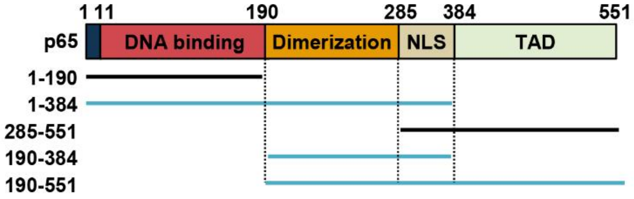

C

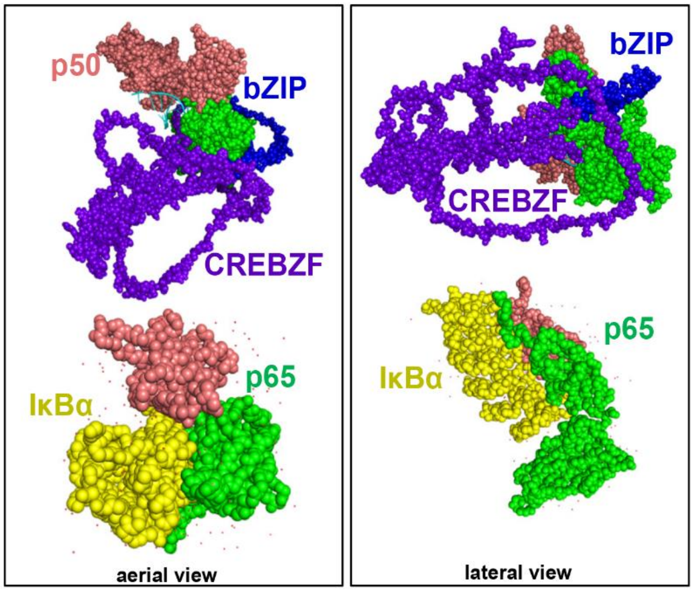

Supplementary Fig. S9

Liu Y, et al.

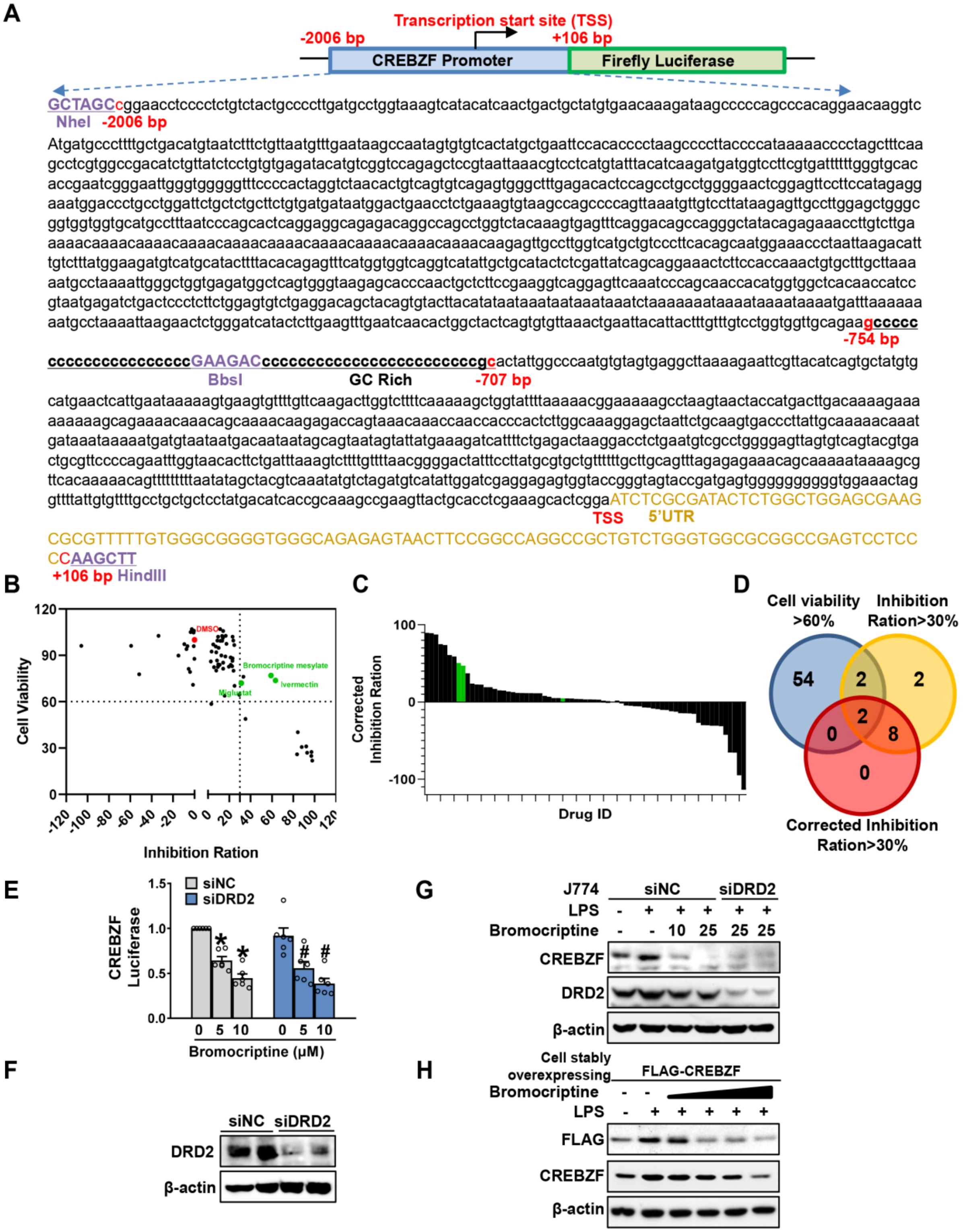

Supplementary Fig. S10

Liu Y, et al.

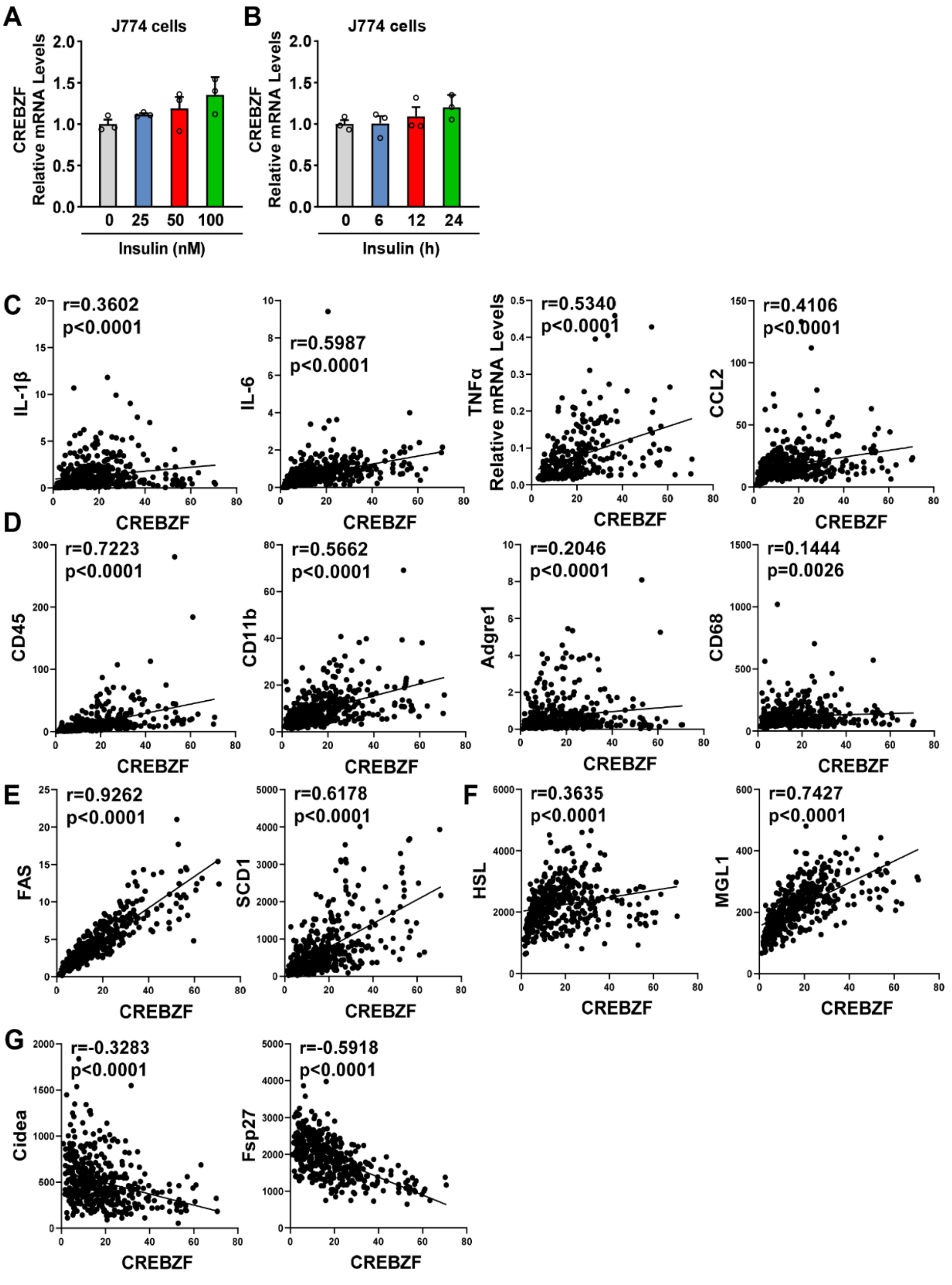

Supplementary Fig. S11 **Liu Y, et al.**

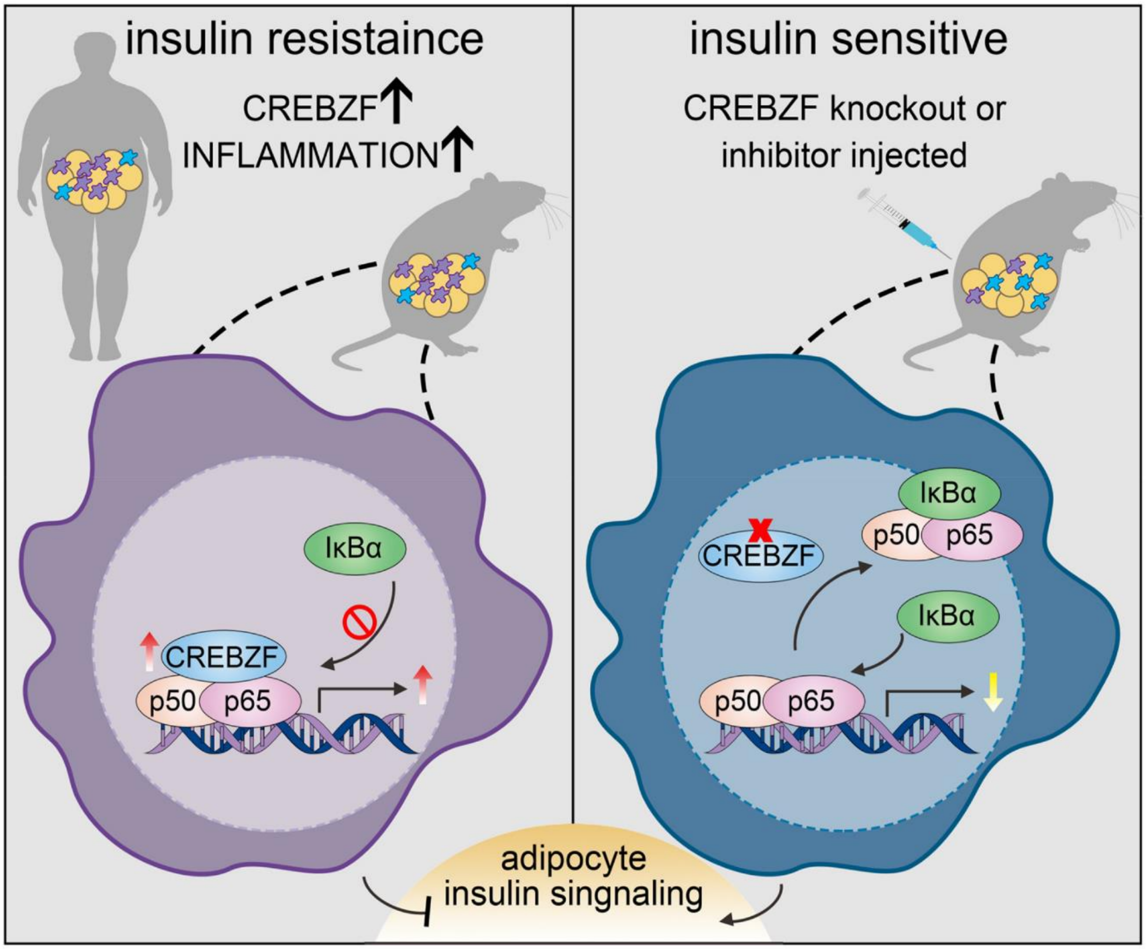

Supplemental Figure Legends

Fig.S1. Myeloid CREBZF deficiency dose not impair immune system development. (A) The percentages of macrophages, neutrophils, and dendritic cells in the bone marrow. (B) The percentages of macrophages and neutrophils in Blood. The percentages of T cell subsets in the thymus (C), lymph nodes (D) and the spleen (E). n = 4. *p<0.05 vs. WT. (F) The protein levels of CREBZF in BMDMs were verified by immunoblotting analysis. Gating strategy of bone marrow (G), thymus (H), blood (I), lymph nodes (J), and spleen (K).

Fig.S2. Myeloid CREBZF deficiency alleviates inguinal adipose tissue inflammation in HFHS-fed mice. Male CREBZF MKO and WT mice at 8 weeks old were fed with a HFHS diet for 16 weeks. (A). Representative flow cytometry analysis and quantification of the $F4/80^{+}CD11b^{+}$ macrophages in SVF from inguinal adipose tissue (iWAT). (B) Representative flow cytometry analysis and quantification of the $CD11c^{+}CD206^{-}$ and $CD11c^{-}CD206^{+}$ subset of total macrophages from iWAT. Chow diet group, n = 6-7. HFHS diet group, n=11-13. *p<0.05 vs. WT mice fed with chow diet; #p<0.05 vs. WT mice fed with HFHS diet. (C) qPCR analysis indicating mRNA abundance of proinflammatory and anti-inflammatory genes in iWAT SVF isolated from CREBZF MKO and WT mice fed with HFHS diet. n = 3-5. (D) The M1/M2 macrophage ratio of eWAT and iWAT from CREBZF MKO and WT mice fed with HFHS diet. n = 11-13. *p<0.05 vs. WT. (E) Gating strategy of macrophages in adipose tissue.

Fig.S3. Differently expressed genes of eWAT from CREBZF MKO and WT mice fed with HFHS diet. (A-B) eWAT from CREBZF MKO and WT mice fed with HFHS diet were performed RNA-seq. n = 3. (A) Heat map representing global differentially expressed genes (DEGs). (B) Heat map representing fibro-related DEGs. Rem, ECM remodeling; Sig, ECM-related signaling. (C) mRNA levels of different immune cells marker genes in SVF from eWAT of CREBZF MKO and WT mice fed with HFHS diet. n = 9-14. *p<0.05 vs. WT mice. (D-E) Myeloid CREBZF deficiency improves HFHS diet-induced hepatic steatosis and insulin sensitivity. (D) Representative H&E staining of liver. Scale bars, 50 μm. (E) Immunoblot analysis of insulin signaling in livers from WT and CREBZF MKO mice fed with HFHS diet.

Fig.S4. Neutrophil depletion in HFHS diet-fed mice. Neutrophil depletion in HFHS diet fed mice were performed by Anti-Ly6G injection. The body weight curve (A) and the final body weight (B) were shown. (C) Neutrophil depletion in eWAT. N = 5. *p<0.05 vs. WT and vehicle. (D) Gating strategy of neutrophils in adipose tissue.

Fig.S5. Myeloid CREBZF deficiency improves adipose tissue inflammation before insulin resistance. Male CREBZF MKO and WT mice at 8 weeks old were fed with a HFHS diet for 8 weeks. (A) Glucose tolerance test (1 g/kg) and respective area under the curve were performed. (B) Insulin tolerance test (1 U/kg) and respective area under the curve were performed. (C) Body weight. (D) Quantifying the $F4/80^{+}CD11b^{+}$ macrophages in SVF from eWAT and the $CD11c^{+}CD206^{-}$ and $CD11c^{-}CD206^{+}$ subset of total macrophages from eWAT n = 11-12. *p<0.05 vs. WT. (E) qPCR analysis indicating mRNA abundance of proinflammatory and anti-inflammatory genes in eWAT SVF isolated from CREBZF MKO and WT mice fed with HFHS diet. n = 7. *p<0.05 vs. WT.

Fig.S6. CREBZF deficiency promotes the IL-4-induced M2-like phenotype.

(A) BMDMs from CREBZF MKO and WT mice were stimulated with IL-4 for 24 h. The percentages of $CD11b^{+}CD206^{+}$ M2-like macrophages were assessed by flow cytometry. n = 2-6. *p<0.05 vs. CREBZF+/+ and IL-4 (B) M2-like marker genes were determined by real-time PCR. n = 4. *p<0.05 vs. CREBZF+/+ and IL-4.

Fig.S7. CREBZF deficiency in macrophages downregulates the NF-κB pathway. Immunoblot analysis in CREBZF+/+ and CREBZF-/- MEF treated 100 ng/ml LPS (A) and 20 ng/ml TNFα (B) as indicated time points. (C) Immunoblot analysis in J774 cells stably overexpressing empty vehicle (EV) or CREBZF treated 100 ng/ml LPS as indicated time points. (D) Immunoblot analysis in CREBZF+/+ and CREBZF-/- BMDMs treated 100 ng/ml LPS as indicated time points. Immunoblot analysis in BMDMs from CREBZF MKO and WT mice treated 100 ng/ml LPS (E), 20 µg/ml poly (I:C) (G), and 20 ng/ml TNFα (H) as indicated time points. (F) Immunoblot analysis in Ad-GFP or Ad-CREBZF infected BMDMs treated 100 ng/ml LPS as indicated time points. (I) Relative mRNA abundance of CREBZF in CREBZF+/+ and CREBZF-/- MEF. n = 4. *p<0.05 vs. WT.

Fig.S8. CREBZF regulates p65. (A) The list of most enriched motifs and transcription factors. The most enriched motifs near peak summits were found by SeqPos tool scanning the JASPAR database. (B) Schematic presentation of p65 domain structures. (C) The protein-protein docking was performed by ClusPro 2.0 and visualized by PyMOL.

Fig.S9. Screening CREBZF inhibitor. (A) Schematic presentation of CREBZF-promoter-luciferase plasmids. The high-throughput screening results of 70 compounds showed cell viability detected by PrestoBlue, inhibition ratio detected by luciferase activities (B) and corrected inhibition ratio (C). (D) Venn showed the distributions of outcomes. (E) The inhibitory effects of bromocriptine mesylate on CREBZF were assessed in stably transfected CREBZF-promoter-luciferase Huh7 cells. DRD2 knockdown in Huh7 cells performed by siDRD2 and treated with bromocriptine mesylate for 48 h. n = 6. *$p<0.05$ vs. siNC and vehicle. (F) DRD2 knockdown efficiency was verified in Huh7 cells. (G) Immunoblot analysis CREBZF protein levels. DRD2 knockdown in J774 cells performed by siDRD2 and treated with bromocriptine mesylate for 48 h. (H) Immunoblot analysis CREBZF protein levels in J774 cells stably overexpressing CREBZF treated with Bromocriptine mesylate for 48 h and 100 ng/ml LPS within the last 4 hours.

Fig.S10. CREBZF is correlated with inflammation and metabolic alterations in human adipose tissues from a database. (A-B) Expression levels of CREBZF are unaltered in response to insulin signaling. J774 cells were treated with insulin in different dosages (A) and a different period (B), and mRNA levels were determined. n=3. (C-G) The set of transcriptome data, including 434 human subcutaneous adipose tissues, were analyzed, the data acquired from GSE135134. The correlations between CREBZF transcriptional levels and markers of proinflammatory cytokines (C), immune cells (D), lipid synthesis (E), lipolysis (F), and lipid droplet protein (G).

Fig.S11. The proposed model for macrophage CREBZF in regulating inflammation and insulin resistance. In response to overnutrition, CREBZF in macrophages is activated and aggravates inflammation leading to impaired insulin sensitivity. CREBZF competitively inhibits the binding of newly

synthesized IκBα to p65, thus enhancing p65 nuclear localization and transcriptional activity. Genetic and pharmacological inhibition of CREBZF attenuates symptoms of type 2 diabetes.

Supplemental Experimental Procedures

Reagents and antibodies—Glucose (cat. G8270), VivoGlo™ Luciferin (cat. P1043) and Dual-Luciferase® (cat. E1980) were purchased from Promega (Madison, WI). Mouse insulin ELISA kit (cat. 10-1249-01) was purchased from Mercodia (Winston Salem, NC). Rabbit monoclonal GAPDH antibody (cat. 5174), rabbit Histone H3 antibody (cat. 9715), mouse myc-tag antibody (cat. 2276S), rabbit NF-κB p65 (cat. 8242), rabbit phospho-NF-κB p65 (Ser536) antibody (cat. 3033), rabbit IκBα antibody (cat. 9242), rabbit phospho-IκBα (Ser32) antibody (cat. 2859), rabbit IKKβ antibody (cat. 8943), rabbit phospho-IKKα/β (Ser176/180) antibody (cat. 2697), rabbit phospho-Akt (Ser473) antibody (cat. 9271) and rabbit monoclonal HA-Tag antibody (cat. 3724) were obtained from Cell Signaling Technology (Beverly, MA). Rabbit polyclonal CREBZF antibody (cat. Ab28700) was purchased from Abcam (Cambridge, UK). APC-anti-CD3 (cat. 17-0032-82), FITC-anti-CD45 (cat. 11-0454-82), PE-anti-F4/80 (cat. 12-4801-82), eFluor 450-anti-CD11b (cat. 48-0112-82), APC-anti-CD206 (cat. 17-2061-80), PE-Cy7-anti-CD11c (cat. 25-0114-81), fixable viability dye eFluor 506 (cat. 65-0866-18) were purchased from eBioscience (San Diego, CA). APC-Cy7-anti-Ly6G (cat. 127623), BV421-anti-CD4 (cat. 100443) and APC-anti-B220 (cat. 103211) were purchased from BioLegend (San Diego, CA). BV650-anti-CD8 (cat. 563234) and mouse Fc block (cat. 553141) were purchased from Becton, Dickinson and Company (Franklin Lakes, NJ). Horseradish peroxidase-conjugated anti-mouse, anti-rabbit, mouse monoclonal β-actin antibody (cat. 69879), anti-goat secondary antibodies; and protein A/G PLUS-Agarose beads were obtained from Santa Cruz Biotechnology (Santa Cruz, CA). Recombinant mouse M-CSF (cat.315-02-100 μg) was purchased from PeproTech (Cranbury, NJ). Recombinant mouse TNFα (cat.410-MT-010) was purchased from R&D Systems (Mineapolis, MN). Invivo MAb anti-mouse Ly6G (BE0075-1) was purchased from Bio X Cell (West Lebanon, NH). Glucose (cat. G8270) and Lipopolysaccharides (L2630-10MG) was purchased from Sigma-Aldrich (St.Louis, MO).

Isolation and differentiation of primary white adipocytes. Subcutaneous fat isolated from 4- to 6-week-old mice was digested for 40-60 min at 37°C with 1 mg/ml collagenase solution. After filtrated and centrifuged, the SVF cells were plated in DMEM supplemented with 10% FBS and 1% P/S. When SVF reached confluence, the cells were passaged. When cells reached confluence again, replaced the medium to induction medium (1 μg/μl insulin, 0.5 mM IBMX, 1 μM dexamethasone and 0.5 μM rosiglitazone) and cultured the cells for 48 h. Then changed to a maintenance medium (1 μg/μl insulin and 1 μM rosiglitazone) for another 7-8 days to obtain mature white adipocytes.

Coculture of primary white adipocytes with BMDMs conditioned media. The protocol is modified from the previously description[2]. BMDMs were treated with 100 ng/ml LPS for 6 h. Then the medium of BMDMs was changed to a serum-free medium. After incubated for another 12 h, the medium was harvested and mixed with fresh serum-free medium at the ratio of 1:1 as BMDMs conditioned media. The mature adipocytes were pretreated with serum-free medium or BMDMs conditioned media for 12 h followed by 100 nM insulin treatment for 15 min or 30 min.

Coculture of white adipose tissues with BMDMs. The protocol is modified from the previously description[3]. Briefly, isolated 0.2 g intact epididymal white adipose tissue from wild-type mice and rinsed it using PBS. Put the tissue into a well in which bone marrow-derived macrophages were pre-cultured. Then treated the cocultured system with 100 ng/ml LPS. After 24 hours, the cocultured system was stimulated by 100 nM insulin for 15 min or 30 min.

Flow cytometry. For flow cytometry analyses of adipose tissue, the protocol is applied as previously described[4]. Mouse eWAT or iWAT was minced into small pieces and digested with 1 mg/ml collagenase type I (Worthington) at 37°C for 45-60 min. After filtrated and centrifuged, the SVF pellets were resuspended in red-blood-cell lysis buffer. For preparing thymic, splenic, and lymph node cell suspensions, the tissues were gently homogenized[5]. Bone marrow cells were flushed with a syringe filled with PBS to isolate cell suspensions from the femurs and tibiae. For whole blood, collected whole blood into heparinized tubes, then added red-blood-cell lysis buffer. Before staining myeloid cells, cells were incubated for 10 min with Fc-block (BD). The fluorochrome-conjugated antibodies including FITC-

anti-CD45, PE-anti-F4/80, eFluor 450-anti-CD11b, APC-anti-CD206, PC-Cy7-anti-CD11c, APC-Cy7-anti-Ly6G, APC-anti-B220, APC-anti-CD3, BV421-anti-CD4, BV650-anti-CD8, FITC-anti-CD44, PE-anti-CD62L. Live/Dead fixable dead cell stain (Invitrogen) was also used. Data were acquired on a CytoFLEX S flow cytometer (Beckman Coulter) and analyzed with CytExpert software (Beckman Coulter).

Metabolic phenotyping. For glucose tolerance tests (GTT), mice were fasted for 16 h followed by intraperitoneal injection with glucose solution (1 g/kg body weight). For insulin tolerance tests (ITT), mice were fasted for 6 h, and intraperitoneally injected with 1 U/kg insulin solution. Blood glucose was determined at 0, 15, 30, 60, 90, and 120 min after injection of glucose or insulin[6]. Triglycerides determination kit (Thermo Fisher Scientific), cholesterol determination kit (Thermo Fisher Scientific), FFA assay kit (Solarbio), and insulin ELISA kit (Millipore) were used for plasma, respectively according to the manufacturer's instructions.

Macrophage chemotaxis assay. BMDMs from WT and CREBZF MKO mice were seeded into the upper wells of a transwell chamber plate (Corning). The macrophages were challenged with 20 ng/ml CCL2 or fresh adipose tissue lysates from obese mice in the lower wells. After incubated for 8 hours, the membrane inserts were fixed and stained by crystal violet. The transmigrated cells on the lower surface of the membrane were imaged by a light microscope and counted by ImageJ software.

ChIP sequencing and bioinformatics analysis. The preparation for ChIP sequencing samples was previously described[7]. BMDMs from CREBZF MKO mice were isolated and infected with adenovirus expressing GFP or myc-tagged CREBZF and followed treatment for 6 hours with LPS. The chromatin was immunoprecipitated by anti-body against myc (Cell Signaling Technology). DNA was then purified by QIAquick columns (QIAGEN) and quantified with Qubit Fluorometer (Thermo Fisher Scientific). ChIP DNA Libraries were prepared by VAHTS® Universal DNA Library Prep Kit for Illumina V3 (Vazyme) according to the manufacturer's protocols. The sequence reads generated by Illumina sequencing were mapped to the mouse genome (mm10) using BWA algorithm, followed by peak calling with MACS2. The differential peaks in promoter regions were identified over the input control, then were analyzed for

determining TF motifs with JASPAR database. And the differential peaks-related genes were analyzed for KEGG pathway analysis by DAVID.

RNA sequencing and bioinformatics analysis. RNA sequencing and data analysis were performed by Majorbio (Shanghai, China). Total RNA was extracted by using TRIzol Reagent (Life Technologies), and the sequencing library was constructed. The expression level of each transcript was calculated according to the transcripts per million reads (TPM) method, then DEGs (differential expression genes) were identified and plotted heatmap. The gene sets were performed GO analysis, KEGG analysis[8], and so forth.

Dual luciferase assays. Cells were co-transfected with plasmids encoding p65 or IκBα as indicated, luciferase reporter plasmids containing different promoter sequences, and Renilla luciferase plasmids as control. 4xNFκB Luc plasmid was purchased from ADDGENE. Renilla luciferase plasmid and pGL3-basic plasmid were purchased (Promega). After 36-48 hours, the activities of Firefly and Renilla luciferase in the lysates were measured with Dual luciferase assays (Promega). The luminescence was measured using an Infinite 2000pro plate reader (TECAN). The results were normalized to Renilla luciferase activity.

Stably transfected CREBZF-promoter-luciferase cell line. To establish stably expressing CREBZF-promoter-luciferase cells, pGL3-CREBZF-promoter and pcDNA3.1 were co-transfected to Huh7. After 24 h, G418 at 800 μg/ml was added for 7 days to select stably transfected cells. Monoclonal cell lines were isolated and further identified by luciferase assay.

High-throughput screening. FDA approved drug library containing 640 compounds (Center for Excellence in Molecular Cell Science, Chinese Academy of Sciences, Shanghai, China) was diluted in DMSO. Stably transfected CREBZF-promoter luciferase Huh7 cells were seeded in 384-well plates by Multidrop Combi (Thermo Fisher Scientific) and treated with 10 μM of each indicated compound. DMSO treatment was used as a control. After 48 hours, the activities of CREBZF promoter were measured by luciferin reactions (Promega) and cell viabilities were measured by Presto Blue (Thermo Fisher

Scientific). During the screening process, the liquid was changed by Handler workstation SX15 (PerkinElmer). The fluorescence was read on EnVision Multimode Plate Reader (PerkinElmer).

Neutrophils depletion. The antibody-mediated neutrophil depletion was performed as described previously[9]. WT and CREBZF MKO mice were fed with a HFHS diet and intraperitoneally injected with anti-Ly6G (BioXCell) thrice a week. For the injection, 100 μg/mouse was used in the first month, and then 200 μg/mouse was used.

Plasmids construction and adenovirus generation. The plasmid encoding HA-tagged p65, myc-tagged p65, FLAG-tagged CREBZF, and FLAG-tagged IκBα was constructed by cloning cDNAs as indicated into the sites of pcDNA3.1 vector. The pGL3-CREBZF-promoter plasmid encoding a luciferase transgene regulated by the CREBZF promoter was constructed by cloning CREBZF promoter into a pGL3-based backbone. Adenoviruses expressing CREBZF or GFP were generated by the AdEasy system as described previously[10, 11].

Lentiviral and stable expression cell lines. Cloning cDNA of CREBZF into pLKO.1 vector and verifying the plasmid expressed successfully. Lentiviral was packaged by using the 3rd generation packaging system in HEK293T. Collected the lentivirus to infect J774 or HEK293T cells. After 48 hours, puromycin was added to select stable expression cell lines.

Cell transfection. Small interfering RNA (siRNA) oligonucleotides were synthesized by GenePharma (Shanghai, China). siRNAs were transfected by Hieff Trans TM in vitro siRNA/miRNA transfection regent (YEASEN). Plasmids were transferred by Lipofectamine 2000 (Invitrogen), and the medium was changed after 8 h. Cells were treated indicated after 24-36 hours and analyzed.

RNA isolation and quantitative real-time PCR analysis. RNA was extracted from tissues or cells by TRIzol Reagent (Life Technologies) according to manufacturer's protocols. Total RNA was then reversely transcribed to cDNA using HiScript III RT SuperMix for qPCR (+gDNA wiper) (Vazyme). qPCR reactions were carried out by ChamQ SYBR qPCR Master Mix (High ROX Premixed) on StepOnePlus Real-Time PCR System (Applied Biosystems)[12].

Immunoblot and immunoprecipitation analysis. The protocol is described previously[12, 13]. Tissues and cells were lysed in NP-40 lysis buffer (50mM Tris-HCl pH 8.0, 150mM NaCl, 1% NP-40, 5 mM EDTA, 1 mM EGTA, 1 mM sodium orthovanadate, 10 mM sodium fluoride, 1 mM phenylmethylsulfonyl fluoride, 2 μg/ml aprotinin, 5 μg/ml leupeptin, and 1 μg/ml pepstatin). Lysates were centrifuged, and the supernatant was detected protein concentration by BCA. Total protein (20-50 μg per lane) was separated by 10% SDS–PAGE and transferred to polyvinylidene difluoride membranes. Membranes were blocked by 5% non-fat milk and incubated with primary antibody followed by horseradish peroxidase-conjugated secondary antibody. The signals were detected by LumiGLO chemiluminescence detection kit (Cell Signaling Technology). For immunoprecipitation, cell lysates were incubated with indicated primary antibodies overnight. And added protein A/G Beads for another 4 hours. The beads were washed three times and analyzed by immunoblots.

Immunofluorescence. Cells were seeded on glass coverslips and treated with 100 ng/ml LPS for 1 h. After washing with PBS and fixing with 4% paraformaldehyde (PFA), the cells were permeabilized with 0.2% Triton X-100 and blocked with 1% BSA (Sigma). Then cells were incubated in primary antibody p65 (Cell Signaling Technology, 1:200 dilution) at 4°C overnight, followed by incubation with goat anti-rabbit Alexa Fluor 488 or Alexa Fluor 594 antibody (Invitrogen, 1:400 dilution). The nuclei were stained by DAPI (Invitrogen), then the coverslips were sealed with nail polish. The fat paraffin sections were cut and mounted on glass slides, then stained with anti-CREBZF antibody (Abcam) and anti-EMR1 antibody (Cell Signaling Technology). Cells and tissues were imaged using a confocal microscope (Confocal Zeiss LSM880).

Histological analysis. Tissues were fixed in 10% phosphate-buffered formalin acetate and embedded in paraffin wax. For hematoxylin and eosin staining, paraffin sections were cut and mounted on glass slides as previously described[13].

Cell lines culture. HEK293T, Huh7, and J774 cells were purchased from Cell Bank, Type Culture Collection Committee, Chinese Academy of Sciences (Shanghai, China). Mouse embryonic fibroblasts (MEF) were derived from CREBZF WT and KO, then immortalization with SV40 T antigen. Cells were

cultured in DMEM supplemented with 10% fetal bovine serum (FBS) and 1% penicillin–streptomycin (P/S) in a humidified 5% CO2 incubator at 37°C as previously described[10].

Protein-protein docking. The crystal structure of the IκBα/NF-κB complex[14] was visualized by PyMOL software. The structure of CREBZF was predicted by AlphaFold. The crystal structure of the p65/p50 heterodimer bound to the DNA complex was studied[15]. CREBZF and the p65/p50 heterodimer were docked by ClusPro2.0, and then the structure was visualized by PyMOL software.

Reference

1. Ying W, Gao H, Dos Reis FCG, Bandyopadhyay G, Ofrecio JM, Luo Z, Ji Y, Jin Z, Ly C and Olefsky JM. MiR-690, an exosomal-derived miRNA from M2-polarized macrophages, improves insulin sensitivity in obese mice. *Cell metabolism*. 2021;33:781-790 e5.
2. Yao J, Wu D, Zhang C, Yan T, Zhao Y, Shen H, Xue K, Huang X, Wang Z and Qiu Y. Macrophage IRX3 promotes diet-induced obesity and metabolic inflammation. *Nature immunology*. 2021;22:1268-1279.
3. He M, Chiang HH, Luo H, Zheng Z, Qiao Q, Wang L, Tan M, Ohkubo R, Mu WC, Zhao S, Wu H and Chen D. An Acetylation Switch of the NLRP3 Inflammasome Regulates Aging-Associated Chronic Inflammation and Insulin Resistance. *Cell metabolism*. 2020;31:580-591 e5.
4. Lumeng CN, Bodzin JL and Saltiel AR. Obesity induces a phenotypic switch in adipose tissue macrophage polarization. *The Journal of clinical investigation*. 2007;117:175-84.
5. Reiley WW, Zhang M, Jin W, Losiewicz M, Donohue KB, Norbury CC and Sun SC. Regulation of T cell development by the deubiquitinating enzyme CYLD. *Nature immunology*. 2006;7:411-7.
6. Gong Q, Hu Z, Zhang F, Cui A, Chen X, Jiang H, Gao J, Chen X, Han Y, Liang Q, Ye D, Shi L, Chin YE, Wang Y, Xiao H, Guo F, Liu Y, Zang M, Xu A and Li Y. Fibroblast growth factor 21 improves hepatic insulin sensitivity by inhibiting mammalian target of rapamycin complex 1 in mice. *Hepatology (Baltimore, Md)*. 2016;64:425-38.
7. Li Y, Wong K, Walsh K, Gao B and Zang M. Retinoic acid receptor beta stimulates hepatic induction of fibroblast growth factor 21 to promote fatty acid oxidation and control whole-body energy homeostasis in mice. *The Journal of biological chemistry*. 2013;288:10490-504.
8. Kanehisa M, Sato Y and Morishima K. BlastKOALA and GhostKOALA: KEGG Tools for Functional Characterization of Genome and Metagenome Sequences. *J Mol Biol*. 2016;428:726-731.
9. Daley JM, Thomay AA, Connolly MD, Reichner JS and Albina JE. Use of Ly6G-specific monoclonal antibody to deplete neutrophils in mice. *Journal of leukocyte biology*. 2008;83:64-70.
10. Li Y, Wong K, Giles A, Jiang J, Lee JW, Adams AC, Kharitonenkov A, Yang Q, Gao B, Guarente L and Zang M. Hepatic SIRT1 attenuates hepatic steatosis and controls energy balance in mice by inducing fibroblast growth factor 21. *Gastroenterology*. 2014;146:539-49 e7.
11. Hu Z, Han Y, Liu Y, Zhao Z, Ma F, Cui A, Zhang F, Liu Z, Xue Y, Bai J, Wu H, Bian H, Chin YE, Yu Y, Meng Z, Wang H, Liu Y, Fan J, Gao X, Chen Y and Li Y. CREBZF as a Key Regulator of STAT3 Pathway in the Control of Liver Regeneration in Mice. *Hepatology (Baltimore, Md)*. 2020;71:1421-1436.
12. Chen X, Zhang F, Gong Q, Cui A, Zhuo S, Hu Z, Han Y, Gao J, Sun Y, Liu Z, Yang Z, Le Y, Gao X, Dong LQ, Gao X and Li Y. Hepatic ATF6 Increases Fatty Acid Oxidation to Attenuate Hepatic Steatosis in Mice Through Peroxisome Proliferator-Activated Receptor alpha. *Diabetes*. 2016;65:1904-15.
13. Li Y, Xu S, Mihaylova MM, Zheng B, Hou X, Jiang B, Park O, Luo Z, Lefai E, Shyy JY, Gao B, Wierzbicki M, Verbeuren TJ, Shaw RJ, Cohen RA and Zang M. AMPK phosphorylates and inhibits SREBP activity to attenuate hepatic steatosis and atherosclerosis in diet-induced insulin-resistant mice. *Cell metabolism*. 2011;13:376-388.

14. Huxford T, Huang DB, Malek S and Ghosh G. The crystal structure of the IkappaBalpha/NF-kappaB complex reveals mechanisms of NF-kappaB inactivation. *Cell*. 1998;95:759-70.
15. Chen FE, Huang DB, Chen YQ and Ghosh G. Crystal structure of p50/p65 heterodimer of transcription factor NF-kappaB bound to DNA. *Nature*. 1998;391:410-3.

Table S1. Characteristics of 44 subjects.

Characteristics	Value	n
BMI (kg/m2)	38.82±7.425	44
Fasting Insulin (pmol/L)	27.42 (22.77-39.87)	41
C-Peptide (pmol/L)	1549±348.7	41
Fasting Glucose (pmol/L)	5.28 (4.53-6.17)	43
HDL-c (mmol/L)	1.065±0.1768	43
LDL-c (mmol/L)	2.992±0.7869	42

Abbreviations: BMI, body mass index; HDL-c, high-density lipoprotein; LDL-c, low-density lipoprotein. Normally distributed data are presented as mean±SD, whereas non-normally distributed data as median with interquartile range.

Table S2. Quantitative RT-PCR primers

Gene	Species	Forward primer	Reverse primer
hCD68	human	CGAGCATCATTCTTTCACCAGCT	ATGAGAGGCAGCAAGATGGACC
hMCP1	human	AGAATCACCAGCAGCAAGTGTCC	TCCTGAACCCACTTCTGCTTGG
hCD163	human	CCAGAAGGAACTTGTAGCCACAG	CAGGCACCAAGCGTTTTGAGCT

hMIP1	human	ACTTTGAGACGAGCAGCCAGTG	TTTCTGGACCCACTCCTCACTG
hβ-actin	human	GATGAGATTGGCATGGCTTT	GTCACCTTCACCGTTCCAGT
hCREBZF	human	AGAAGGAGTACGTGATGGGG	CGTAGGTAGCGACTCTCCTC
mCREBZF	mouse	TAATCGGCTCAAGAAGAAGG	CGTAGGTAGCGACTCTCC
mβ-actin	mouse	CCACAGCTGAGAGGGAAATC	AAGGAAGGCTGGAAAAGAGC
mAdgre1	mouse	TTGTACGTGCAACTCAGGACT	GATCCCAGAGTGTTGATGCAA
mMertk	mouse	CAGGGCCTTTACCAGGGAGA	TGTGTGCTGGATGTGATCTTC
mIL-6	mouse	CCACGGCCTTCCCTACTTCA	TGCAAGTGCATCATCGTTGTTC
mIL-1β	mouse	TACCAGTTGGGGAACTCTGC	CAAAATACCTGTGGCCTTGG
mTNFα	mouse	GAACTGGCAGAAGAGGCACT	AGGGTCTGGGCCATAGAACT
mNos2	mouse	TGTTAGAGACACTTCTGAGGCTC	CACTTTGGTAGGATTTGACTTT
mArg1	mouse	AAGACAGCAGAGGAGGTGAAGAG	TGGGAGGAGAAGGCGTTTGC
mMrc1	mouse	CAAGGAAGGTTGGCATTTGT	CCAGGCATTGAAAGTGGAGT
mMgl1	mouse	CTGGATCCTGGTGTCTTGGT	AGGTGGGTCCAAGAGAGGAT
mYm1	mouse	CTTCCACACAGGAGCAGGAATC	GCTCCATGGTCCTTCCAGTA
mCD117	mouse	GAGTTCCATAGACTCCAGCGTC	AATGAGCAGCGGCGTGAACAGA
mCD19	mouse	GCCACAGCTTTAGATGAAGGCAC	CATCCACCAGTTCTCAACAGCC
mCD56	mouse	GGTTCCGAGATGGTCAGTTGCT	CAAGGACTCCTGTCCAATACGG
mCD4	mouse	GTTCAGGACAGCGACTTCTGGA	GAAGGAGAACTCCGCTGACTCT
mCD8	mouse	ACTACCAAGCCAGTGCTGCGAA	ATCACAGGCGAAGTCCAATCCG
mLy6G	mouse	AGAAGCAAAGTCAAGAGCAATCTCT	TGACAGCATTACCAGTGATCTCAGT
mFoxp3	mouse	CCTGGTTGTGAGAAGGTCTTCG	TGCTCCAGAGACTGCACCACTT

Table S3. siRNA sequences

Gene	Species	Forward primer	Reverse primer
siDrd2	human	AAUGUAGAUCUUGAUGUAGAC	CUACAUCAAGAUCUACAUUGU
siDrd2	mouse	AAUGUUGAAGGUGGUAUAGAU	CUAUACCACCUUCAACAUUGA
siIκBα	mouse	GGACGAGGAGUACGAGCAAAU	UUGCUCGUACUCCUCGUCCUU
